# Unlocking the door for synergizing expansion–capacity in Si anodes: breathable flexible MOFs

**DOI:** 10.1039/d6sc05688a

**Published:** 2026-08-11

**Authors:** Wenhao Zhou, Fan Mo, Yitong Wang, Xinrui Mu, Haibo Li

**Affiliations:** a School of Resources and Civil Engineering, Northeastern University Shenyang 110819 China lihaibo@mail.neu.edu.cn

## Abstract

The trade-off dilemma between volume expansion and capacity limits the application of Si-based anodes in Li-ion batteries. Breathable flexible metal–organic frameworks (MOFs) provide a viable strategy to tackle this challenge. They can relieve lithiation-induced mechanical stress *via* reversible coordination deformation of metal nodes and organic ligands, while maximizing the retention of specific capacity. This review first elaborates the lithium storage mechanism of silicon and compares traditional volume suppression strategies, proving excessive expansion inhibition inevitably sacrifices specific capacity. MOF modifiers are divided into breathable flexible MOFs, rigid MOFs and MOF-derived carbons, and their differentiated mechanisms for mitigating volume expansion and stabilizing SEI films are comprehensively elucidated. Four core preparation optimization strategies including conductivity compensation, interfacial bonding reinforcement, ligand engineering, crystal engineering are summarized. Major bottlenecks such as unclear node–ligand matching rules, high manufacturing cost, low volumetric energy density and insufficient quantitative characterization of MOF breathing behavior are outlined. Corresponding research priorities covering quantitative structure–property correlation, non-destructive encapsulation and *in situ* dynamic characterization are put forward. This review provides a theoretical basis and technical guidance for the scalable application of high-performance silicon anodes in LIBs.

## Abstract 2

The trade-off dilemma between volume expansion and capacity limits the application of Si-based anodes in Li-ion batteries.

## Graphical abstract

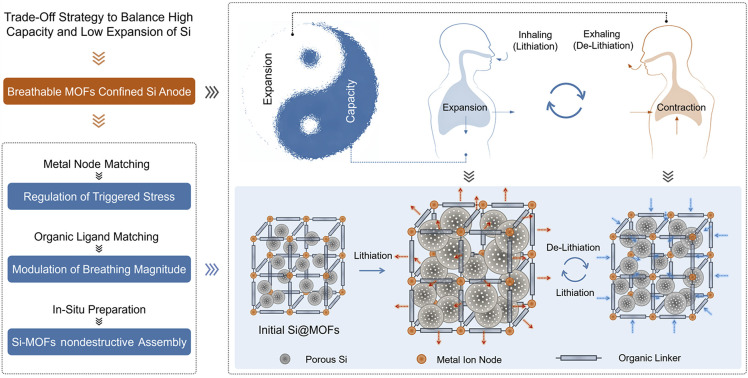

## 1. Introduction

As the cornerstone of modern energy storage, lithium-ion batteries (LIBs) are widely employed in portable electronics, electric vehicles and grid-scale energy storage.^1,2^ However, with the rapidly rising demand for high-energy-density batteries, graphite anodes (only 372 mAh g^−1^) have become a critical capacity bottleneck, creating an urgent need for high-capacity alternatives.^3^ Silicon-based anodes exhibit a theoretical capacity of 4200 mAh g^−1^, making them highly promising candidates.^4,5^ However, their volume expansion upon lithiation exceeds 300%, causing electrode damage, poor cycling stability and low coulombic efficiency, thus hindering commercialization.^5–8^ Meanwhile, excessive suppression of expansion causes severe capacity loss.^9,10^ Thus, an inherent contradiction exists between capacity and expansion.

To address the inherent “high capacity–low expansion” paradox in silicon-based materials, existing research has developed a range of regulatory strategies. At the level of material structural design, nanoengineering (*e.g.*, nanoparticles and nanowires) or porous architecture construction efficiently mitigates stress concentration-induced volume expansion.^11–13^ At the interface engineering level, carbon coating and metal oxide modification are employed to fabricate buffer layers, which alleviate volume expansion-driven material structural damage and preserve the integrity of the solid electrolyte interface film.^14,15^ System-level optimization strategies, such as pre-lithiation compensation for lithium loss, can indirectly mitigate the volume expansion of silicon materials.^16,17^ These strategies are geared, either directly or indirectly, toward mitigating volume expansion to bolster cycling stability, yet they often overlook the retention of high specific capacity.^18,19^ Therefore, targeted and systematic strategies are needed to balance high electrochemical capacity and structural stability.^20–22^

Metal–organic frameworks (MOFs) are crystalline porous materials assembled *via* coordination of metal ions/clusters with organic ligands.^23^ MOFs feature high structural tunability, versatile functions and tunable interfacial properties. Certain MOFs exhibit a distinctive breathing effect: their frameworks switch reversibly between closed and open pore states during Li^+^ insertion and extraction, achieving a 10–50% reversible change in pore volume without structural collapse.^24–27^ As a typical example, MIL-53 is constructed from one-dimensional rigid inorganic chains interconnected by µ-OH–bridged octahedral Al^3+^/Fe^3+^ metal nodes, which serve as the framework's stress-bearing backbone with no covalent bond rupture throughout deformation. In the intrinsic closed-pore configuration, contracted M–O-carboxylate bond angles narrow the spacing between metal–oxygen chains. During lithiation, joint external forces generated by silicon swelling and pore-trapped lithium ions twist M–O coordination angles by 12–34° and slightly shift metal centers laterally along the channels;^24,28,29^ µ-OH bridges stretch minimally while all M–O covalent bonds remain intact, and the inorganic chains only translate as a whole. After delithiation, stress vanishes, coordination angles rebound spontaneously and the entire framework recovers its original geometry. Metal nodes merely supply structural rigidity, and all reversible framework expansion and contraction stem from bond-angle torsion at node–linker joints.^26,30^ This adaptive dynamic structure well accommodates the drastic volume fluctuation of silicon anodes, providing a new strategy to reconcile their high specific capacity and severe volume expansion.^31^ For example, a novel flexible MOF with a deformable Mn_8_ cluster has been reported, which exhibits intelligent guest-responsive breathing through cluster contraction and ligand migration, enabling highly selective separation of light hydrocarbons and Freon gases.^32^ Similarly, a water-processable Mg-MOF is reported that undergoes reversible coordination reconstruction to tune its aggregation state, thereby realizing switchable luminescence properties for ink-based printing, anti-counterfeiting and optical devices.^33^ Specifically, its core advantage manifests in the synergistic effect across three dimensions. First, the structural designability of MOFs provides precise spatial control. By tuning organic ligand length and metal nodes, pore size (0.3–10 nm) and framework elasticity can be tailored to endow the frameworks with reversible breathing behavior and form effective buffer spaces,^34,35^ mitigating silicon's volume expansion and structural stress during lithiation.^36,37^ Second, the functional versatility of MOFs synergistically optimizes electron transport and catalytic activity. Upon high-temperature pyrolysis, flexible MOFs with an intrinsic breathing effect are converted into porous carbon that integrates mechanical buffering, high conductivity and rich active sites. This material mitigates the low conductivity of silicon anodes, while residual metal sites accelerate lithium ion migration and interfacial reaction kinetics. Although high-temperature treatment disrupts the original flexible skeleton and impairs the breathing effect to form a rigid structure, the enhanced conductivity and optimized reaction kinetics dominate, enabling a favorable dynamic balance between capacity retention and volume expansion suppression.^38,39^ Third, interfacial modulation stabilizes SEI formation. The surface functional groups and Lewis acid–base metal sites of MOFs interact with fluorinated electrolyte components, forming a stable LiF-rich SEI layer on silicon-based electrodes. This layer features high mechanical strength and ionic conductivity.^40,41^ It withstands volume expansion stress and suppresses electrolyte side reactions.^42^ These synergistic effects could potentially enable MOFs to precisely balance volume expansion and specific capacity in silicon-based anodes. Nevertheless, a systematic summary of the relevant mechanisms and the development of targeted optimization strategies have not yet been well established.

In recent years, research on Si@MOF composites derived from MOFs has advanced from simple composite fabrication to precise “structure–function” engineering, with the core objective consistently focusing on resolving the trade-off between high capacity and volume expansion in silicon-based materials. Based on the framework structure and derived morphology, the composites are divided into three categories. Breathable/gated MOFs (*e.g.*, MIL-53) have dynamically reversible frameworks. Their breathing effect drives reversible pore expansion and contraction to elastically mitigate silicon volume expansion.^43,44^ Rigid MOFs feature robust frameworks with excellent stability and conductivity but no dynamic responsiveness; they only work *via* physical confinement and cannot adapt to the drastic deformation of silicon electrodes.^44,45^ Carbon-based derivatives are obtained by high-temperature pyrolysis of the two MOF types, which deliver physical buffering through porous structures and achieve greatly improved conductivity.^39^

This review systematically illustrates the key bottlenecks of silicon anodes for lithium-ion batteries, discusses their alloying-based lithium storage mechanism, and describes the progressive structural degradation induced by lithiation-driven volume expansion. After summarizing conventional modification approaches, we identify their common drawback: these strategies only realize unidirectional volume control and tend to sacrifice capacity for long-term cycling stability. Emphatically focusing on flexible MOFs with an intrinsic breathing effect, we thoroughly discuss their modification principles and composite engineering, including conductive and catalytic functional designs. A unique three-dimensional regulation mechanism of “buffering-conduction-interface” is further established. On this basis, we propose a trade-off strategy based on breathable MOFs to resolve the inherent conflict between capacity and volume expansion, which paves the way for large-scale application of high-performance silicon anodes.

## 2. Mechanisms of lithium storage and volume expansion in silicon-based materials

Silicon-based anode materials, featuring a unique alloy-based lithium storage mechanism, exhibit an ultrahigh theoretical specific capacity of 4200 mA h g^−1^.Si + *x*Li^+^ + *x*e^−^ ↔ Li_*x*_Si, 0 ≤ *x* ≤ 4.4

**Fig. 1:**
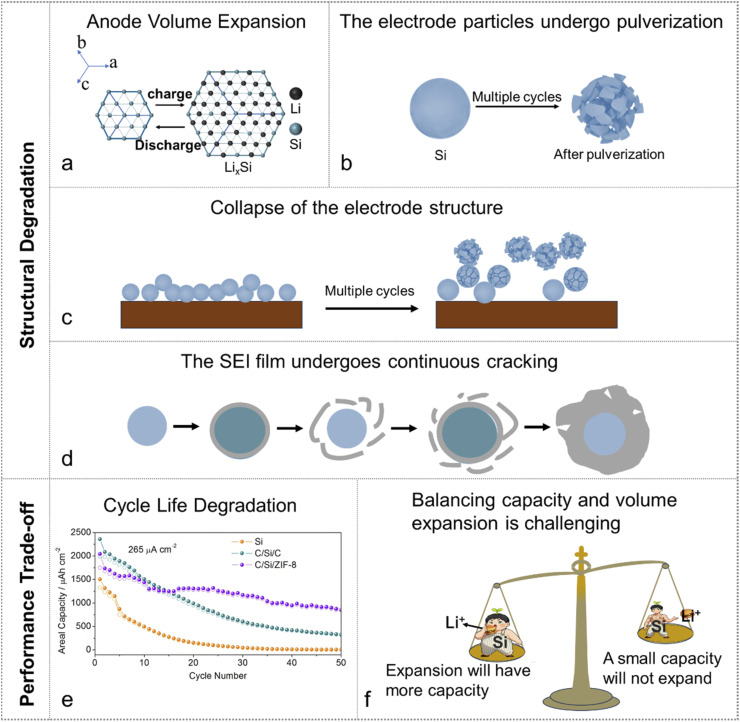
This figure systematically summarizes the fundamental principles and core challenges faced by silicon-based lithium-ion batteries. (a) Structural evolution of silicon anodes during lithiation/delithiation, illustrating volume expansion from Li–Si alloying. (b) Pulverization of silicon particles after cycles, a critical failure mechanism from volume expansion.^52^ Adapted/reproduced from ref. 52 with permission from Royal Society of Chemistry, copyright 2017. (c) Post-cycle electrode structure collapse, where particle pulverization and expansion impair integrity and electrical contact. (d) Repeated cracking and repair of the SEI layer on silicon particles during volume expansion/shrinkage, causing electrolyte consumption and capacity fade. (e) Capacity–cycle curves of different materials, showing poor cycling stability of pure silicon anodes.^53^ Adapted/reproduced from ref. 53 with permission from American Chemical Society, copyright 2015. (f) A balance metaphor to highlight the core trade-off: high silicon capacity comes with severe expansion, while low-expansion materials lack sufficient capacity.

These materials far outperform graphite anodes (372 mAh g^−1^) that rely on an intercalation mechanism to form LiC_6_ (6C atoms per Li atom). Their high capacity is derived from the deep alloying reaction between Si and Li, where each Si atom can bind up to 4.4 Li atoms to form Li_*x*_Si alloy phases.^3,5^ However, as shown in Fig. 1a, the lithiation process leads to significant changes in the crystal structure. At room temperature, diamond-cubic Si (5.43 Å) sequentially transforms into Li_12_Si_7_, Li_7_Si_3_, Li_13_Si_4_, and finally into the thermodynamically stable Li_15_Si_7_ phase (10.685 Å) while at elevated temperatures, it further converts to Li_4.4_Si. With an overall lattice expansion of 97%, Table 1 shows the theoretical capacities and volume expansion rates of different Li–Si alloy phases; more Li^+^ intercalation leads to severe volume expansion of the material.^46–49^ During charge–discharge cycling, this drastic volume change induces multiscale cascading damage: the electrode undergoes cyclic stresses up to 1.4 GPa, causing irreversible Si pulverization (micron-sized particles reduce to the nanoscale within a few cycles, Fig. 1b) and eventual collapse of the entire electrode structure, as shown in Fig. 1c.^22,50^ The SEI repeatedly fractures and regenerates, forming a thick, brittle Li_2_CO_3_-based layer that induces an initial irreversible capacity loss of 20–40%. As shown in Fig. 1d, the fragmented SEI often wraps around silicon particles, forming “dead silicon”.^7,8,51^

**Table 1:** Theoretical capacity and volume expansion rate of different Li–Si alloy phases

Li–Si alloy phase	*x* in Li_*x*_Si	Theoretical capacity, mAh g^−1^	Volume expansion, %
Si	0	0	0
LiSi	1	954	160
Li_12_Si_7_	1.71	1635	222
Li_2_Si	2	1900	263
Li_13_Si_4_	3.25	3100	389
Li_15_Si_4_	3.75	3590	390
Li_22_Si_5_	4.4	4200	420

The drastic volume variation damages the carbon black/PVDF conductive network, disrupting electron transport and shortening cycle life. Fig. 1e presents the long-cycle performance of Si, C/Si/C, and C/Si/ZIF-8. Unmodified Si exhibits the most severe capacity decay, while C/Si/C and C/Si/ZIF-8 show remarkable improvement. This enhancement is likely attributed to their effective suppression of Si volume expansion and preservation of the conductive network, although the underlying mechanism requires further investigation.^7,54^ The aforementioned issues highlight the core contradiction of silicon-based anodes: the trade-off between high specific capacity and severe volume expansion. Fig. 1f vividly illustrates this relationship. Restrictive strategies (*e.g.*, stacking pressure regulation) can mitigate the drastic volume expansion induced by high capacity, suppress structural degradation and retain energy density, yet excessive restriction reduces electrode porosity, impedes lithium-ion transport and thus compromises specific capacity.^9,54,55^

## 3. Conventional volume expansion mitigation strategies and their limitations

Despite being core candidates to replace traditional graphite owing to their high specific capacity, Si-based anodes face commercialization barriers. There exists an inherent contradiction between the severe lithiation-induced volume expansion of Si and the capacity bottleneck of graphite, which is further compounded by the application limitations of current regulation strategies. Through a comparison of representative materials, cycling performance, volume expansion rates, and key aspects of existing strategies, accordingly, the following sections will identify potential improvement directions for current silicon-based anode technologies.

### 3.1 Crystallinity engineering

**Fig. 2:**
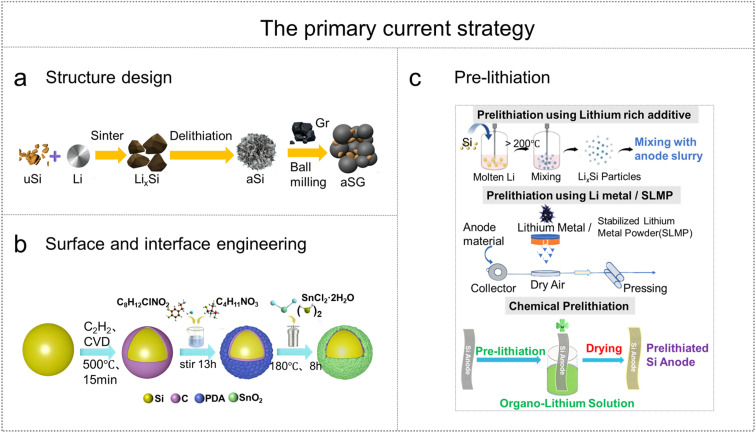
(a) The fabrication of an amorphous silicon–graphite composite (aSG) from micron-sized silicon (uSi) *via* lithium sintering, delithiation, and ball milling.^11^ Adapted/reproduced from ref. 11 with permission from Elsevier, copyright 2025. (b) The multi-layer coating modification of silicon particles: a carbon layer is deposited *via* CVD, followed by PDA interface modification and SnO_2_ deposition, forming a core–shell structure to enhance interface compatibility and suppress side reactions.^14^ Adapted/reproduced from ref. 14 with permission from American Chemical Society, copyright 2024. (c) Diverse pre-lithiation techniques (*e.g.*, lithium additive, molten lithium, and chemical pre-lithiation) to boost initial coulombic efficiency and cycling stability.^56^ Adapted/reproduced from ref. 56 with permission from Institute of Physics, copyright 2023.

Material structural design mitigates volume expansion stress at its source by regulating the morphology and aggregation state of silicon. Representative strategies encompass nanoscale modification and composition structure design. For example, the “solid-state amorphous” strategy by Song *et al.* (2025) is highly representative. *Via* structural engineering, isotropic amorphous Si (A-Si) replaces crystalline Si and is combined with graphite at a specific ratio to form aSG60. The corresponding technical route is presented in Fig. 2a. Crystalline silicon (c-Si) with regular crystal orientation undergoes lattice transformation accompanied by atomic arrangement disparities during lithiation, causing uneven local volume variation, extreme stress concentration, and ultimately anisotropic expansion. In contrast, amorphous silicon (a-Si) features an amorphous structure without distinct crystal orientation, exhibiting isotropic stress–strain behavior. During lithiation, Li^+^ intercalates uniformly, enabling overall homogeneous volume expansion free of local stress accumulation, which fundamentally mitigates the “local overexpansion and stress concentration” issue of c-Si, converting the volume change from “non-uniform and destructive” to “uniform and controllable”. Leveraging the synergy of A-Si's isotropic expansion and porous structure, stress concentration is fundamentally alleviated, and after 50 cycles, aSG60 exhibits a volume expansion of only 38%. At 0.1 A g^−1^, it delivers a reversible capacity of 1540.1 mAh g^−1^ with 84.6% initial coulombic efficiency and shows capacity retentions of 86% (100 cycles at 0.5 A g^−1^) and 70% (200 cycles at 1 A g^−1^). It effectively suppresses volume expansion while retaining the high capacity and long-cycle stability of Si-based anodes, balancing the trade-off between lithium storage capacity and volume expansion to a certain extent. However, precise regulation of the aSi–graphite ratio remains a core challenge: excessive aSi induces a drastic decline in cycle stability, while insufficient aSi fails to fully exploit its high-capacity merit, making it challenging to strike the ideal balance between “extreme capacity” and “ultra-long cycling”.^11^ This makes effective control of volume expansion difficult. Similarly, a group from Sungkyunkwan University (South Korea) fabricated a reticulated nanosilicon structure using anodized aluminum as a template. First, this strategy utilizes amorphous silicon, which undergoes no distinct phase transition during lithiation/delithiation. Additionally, its three-dimensional voids accommodate lithiation-induced expansion and relieve stress, effectively mitigating volume expansion. Its capacity retention reached 97.6% after 200 cycles, demonstrating excellent structural stability. However, the embossed silicon nanomesh exhibits an initial coulombic efficiency of only 52.3%, insufficient volumetric energy density, slight pulverization after multiple cycles, and a long-term risk of pore collapse.^12^ While strategies including amorphization, Si–graphite composites, nanosizing, and porous structures offer notable passive buffering effects toward the volume expansion of Si anodes, further optimization is still required to fully address the intrinsic expansion characteristics of Si. These approaches also face trade-offs between capacity and cycling stability, along with persistent challenges related to structural stability and volumetric energy density, which represent key hurdles for their practical deployment.

### 3.2 Interfacial engineering

Coating engineering mitigates electrode structural damage from volume expansion by constructing protective layers and regulating SEI film stability. For example, the core–shell Si@NC@SnO_2_ designed by Assoc. Prof. Li Xiaodan's group (Xiamen Univ. of Technol.) has an outer protective layer uniformly coating the nanosilicon core. Fig. 2b depicts the preparation of the Si@C@PDA@SnO_2_ multilayer core–shell Si-based anode. A conductive carbon layer is deposited *via* CVD, a PDA interfacial layer is formed by self-polymerization, and a SnO_2_ capacity layer is introduced hydrothermally. The PDA layer regulates the interface, reinforces interlayer adhesion, stabilizes the SEI film, suppresses particle agglomeration, and buffers volume expansion. Combined with the high conductivity of carbon and capacity compensation of SnO_2_, this structure effectively restrains Si volume expansion, balances high capacity and long cycling stability, and significantly improves the anode performance.^14^ At 0.3 A g^−1^, the half-cell electrode delivered a reversible specific capacity of 1056 mA h g^−1^ after 200 cycles with longer cycle life than pure Si electrodes, verifying the coating strategy's effectiveness. Nevertheless, the initial coulombic efficiency is around 72.5%, and further optimization of its capacity and cycling stability would be beneficial for advancing its practical application. Another study constructed a high-strength ordered encapsulated structure consisting of SiO_*x*_/hard carbon/closed-cell carbon multi-layer coated Si quantum dots. Using SiO_*x*_ to enhance interfacial bonding, hard carbon to buffer expansion stress, and closed-cell carbon to block electrolyte erosion, its capacity retention exceeded 70% after 500 cycles at 5 A g^−1^ (high current density). This confirms that the coating strategy significantly enhances structural stability.^15^ In addition, repeated SEI fragmentation and regeneration is a core mechanism of Si anode capacity fading; thus, regulating SEI stability is another pivotal direction in interface engineering. For example, one study constructed a uniform, dense, highly conductive, and high mechanical strength SEI film dominated by LiF and Li_2_C_2_O_4_*via* an intermittent discharge strategy. Intermittent discharge eliminates LiDFBOP's soluble product accumulation, fosters inorganic-rich SEI precursors (*e.g.*, LiF), optimizes the SEI into a uniform, dense structure with high mechanical strength, and lowers Li^+^ diffusion barriers for uniform lithiation/delithiation, yielding a stable SEI layer. This enables uniform Li^+^ intercalation/deintercalation to avoid local high stress, buffer volume variations, inhibit particle fragmentation and SEI re-growth, and ultimately reduce the average thickness of silicon-based anodes by 14.2% after 500 cycles *versus* the unmodified group and realizing volume expansion suppression.^57,58^ Nevertheless, most of these strategies tend to focus more on effectively suppressing volume expansion through structural or interfacial stabilization, while the rational balance between achievable capacity and expansion mitigation still requires further delicate coordination and optimization.

### 3.3 Prelithiation engineering

Beyond the aforementioned mainstream research perspectives, current studies also focus on optimizing the Li-ion battery system to suppress Si-based material volume expansion during charge–discharge cycles and prevent expansion-induced adverse consequences.

For example, prelithiation technology effectively compensates for initial coulombic efficiency loss of Si-based materials and synergistically enhances electrode performance in cycling stability and capacity retention. Fig. 2c shows part of the pre-lithiation technology; pre-lithiation includes four types: direct lithium metal, stable lithium metal powder, lithium-silicon alloy, and chemical pre-lithiation. It raises the initial coulombic efficiency of silicon anodes from 50–80% to over 85%, suppresses side reactions, mitigates volume expansion, and significantly improves battery performance.^56^ For example, Ji *et al.* prepared prelithiated Si *via* lubricant-assisted ball milling, and its Li_*x*_Si–Li_*x*_Si symmetric battery achieved stable cycling over 320 h at 4 mAh cm^−2^.^17^ Kim *et al.* developed a dry pre-lithiated diffusion-dependent electrode (PL-DDE) with Li metal powder. The optimal 3 wt% PL-DDE raises initial coulombic efficiency by 33%, 200-cycle average coulombic efficiency and capacity retention by 1% and 20.1% in half-cells, and by 11.5 and 15.7% in full-cells.^59^ The underlying mechanism lies in the compensation of active lithium loss and the *in situ* formation of a stable SEI film by the preconstruction of the Li_*x*_Si alloy phase, which jointly alleviate volume expansion, reduce side reactions, and enhance structural integrity during cycling.^59,60^

In summary, although prelithiation strategies can alleviate the volume expansion of Si-based anodes to a certain degree, they still cannot fully address the intrinsic volume change issue at a fundamental level. In particular, these approaches often focus on relieving or buffering mechanical stress, rather than adopting a more balanced strategy centered on effective stress absorption. Moreover, the relatively high costs associated with these techniques may also limit their further practical application.

## 4. The application prospects of MOFs in silicon-based electrodes

**Fig. 3:**
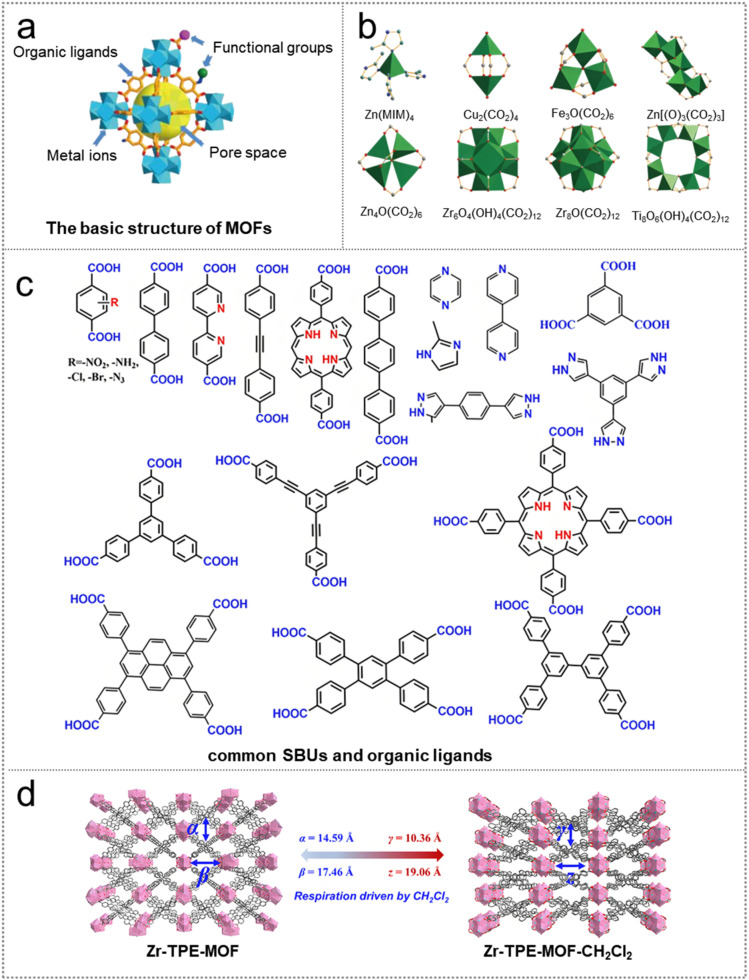
(a) Core components of MOFs (metal ions, ligands, functional groups, and pores).^64^ Adapted/reproduced from ref. 64 with permission from Elsevier, copyright 2019. (b) Typical metal-oxide carboxylate SBUs.^64^ Adapted/reproduced from ref. 64 with permission from Elsevier, copyright 2019. (c) Common carboxylic acid/nitrogen-containing ligands and SBUs.^64^ Adapted/reproduced from ref. 64 with permission from Elsevier, copyright 2019. (d) Structural “breathing” of Zr-TPE-MOF upon CH_2_Cl_2_ uptake, showing altered lattice parameters.^62^ Adapted/reproduced from ref. 62 with permission from Wiley-VCH Verlag, copyright 2025.

MOFs provide an effective route to address issues of Si-based materials. With flexible structure, versatile functions and superior physicochemical properties, MOFs can effectively reconcile the inherent conflict between volume expansion and high capacity in Si anodes. Notably, their breathable structural feature enables adaptive expansion and contraction upon lithiation–delithiation, in which the flexible coordination bonds can reversibly deform to accommodate large volume swings without structural collapse.^61,62^ Meanwhile, the porous framework effectively absorbs mechanical stress, the conductive moieties construct efficient electron transport pathways, and the highly tunable chemical environment helps stabilize the SEI and improve interfacial compatibility.^63^ Structural diversity allows precise matching with Si electrodes. Fig. 3a schematically illustrates the structure of functional MOFs, which are constructed from metal nodes (inorganic coordination centers) and organic linkers that self-assemble into a 3D porous framework *via* coordination bonds. MOFs feature permanent porosity and tailorable functional groups (*e.g.*, carboxyl, amino, and pyridyl) on ligand termini. Functional groups such as pyridyl and amine can be directly incorporated into linkers for molecular recognition, while multivariate MOFs and post-synthetic modification enable the introduction of multiple functional sites and synthesis-incompatible groups, respectively. Tuning metal/ligand combinations imparts tailored electronic, magnetic, and optical properties, and their tunable pore sizes (from microporous to macroporous) allow hosting diverse guests (*e.g.*, single-atom metals, nanoparticles, and enzymes), greatly expanding functionalities beyond pristine MOFs.^64,65^

Fig. 3b and c display typical secondary building units (SBUs) and organic ligands of MOFs. Most organic ligands are carboxylic acid or nitrogen-containing moieties, which endow MOFs with excellent structural and functional tunability. Deng, H. *et al.* systematically extended ligand lengths to construct the isoreticular IRMOF-74-n series, validating the superior designability of MOFs.^66^ Notably, some MOFs exhibit a unique breathing behavior, in which their pore size and structure dynamically respond to external guest molecules.

As presented in Fig. 3d, the pore size of Zr-TPE-MOF undergoes a distinct change upon uptake of CH_2_Cl_2_ vapor, with the pore length and width varying from 14.59 and 17.45 Å to 10.36 and 19.06 Å, respectively. This structural alteration stems from the fact that the incorporated CH_2_Cl_2_ disrupts the motion of molecular rotors and triggers a change in the unit cell structure. This feature offers key optimization for accommodating volume expansion of Si-based anodes during lithiation.^62^ Therefore, by rationally tuning metal nodes and organic ligands, numerous MOFs with tailored pore sizes and versatile functionalities (*e.g.*, conductivity, catalytic activity, confinement effects, and breathing behavior) can be created, providing abundant material options to balance volume expansion and high lithium-storage capacity for Si-based anodes.^64^

### 4.1 Classification and mechanism of action of Si@MOFs

MOFs are classified into three categories based on framework dynamics and derived forms: breathable/gate-opening MOFs, rigid MOFs, and derivatives of MOFs. Breathable/gate-opening MOFs feature dynamically reversible frameworks and a distinct breathing effect, enabling reversible pore expansion and contraction during Li^+^ intercalation/deintercalation. Such a feature adaptively buffers the severe volume expansion of silicon anodes while preserving high lithium-storage capacity, rendering them highly promising for balancing capacity and volume expansion in silicon anodes. Rigid MOFs possess static, robust frameworks with exceptional thermal, chemical, and mechanical stability as well as higher conductivity, yet lack dynamic responsiveness. They only alleviate volume expansion through a single confinement effect and cannot adapt to the large deformation of silicon electrodes.^44,45,61^ Upon high-temperature pyrolysis, both MOF types are converted into rigid carbon-based materials, with the breathing effect fully eliminated; these derivatives provide merely physical buffering *via* porous structures but exhibit remarkably improved conductivity.^67^ Hereafter, a detailed comparison of the three categories is conducted.

#### 4.1.1 Breathable/gate-opening MOFs

Breathable MOFs undergo reversible pore evolution *via* flexible coordination to form elastic voids, buffering volumetric fluctuation of silicon during lithiation/delithiation. Integrating dynamic buffering and spatial confinement, their tunable pores and metallic Lewis sites accelerate Li^+^ transport and generate a robust LiF-rich SEI, while metal–ligand modulation tailors framework elasticity for efficient stress dissipation.^40–42^ Although gate-opening MOFs cannot undergo reversible two-phase transformation between narrow-pore and large-pore polymorphs, reversible linker rotation enables continuous pore regulation and framework self-adaptive buffering. In contrast to rigid MOFs with invariant pore configuration and non-deformable skeleton, they alleviate partial volume stress of silicon anodes *via* gradual aperture opening/closing and deliver favorable reversible elastic buffering capacity. Unlike conventional strategies that restrain expansion at the expense of capacity, such MOFs offer an effective strategy to resolve silicon's inherent capacity–expansion trade-off.^44^

MIL-53(Al/Fe/Cr), a classic flexible breathing MOF built from [M–OH] chains and terephthalate ligands with 1D rhombic channels, undergoes thermodynamically reversible pore switching *via* tunable bond angles and guest hydrogen bonding; the three analogs differ only in phase-transition conditions and kinetics with identical breathing nature.^43,68^ Researchers synthesized rod-like MIL-53(Al) *via* two-step hydrothermal treatment and fabricated three anodes: Gr/Si-M (5 wt% MIL-53(Al)), Gr/Si–S (5 wt% Super P) and bare Gr/Si by dry coating. At 0.3C over 100 cycles, Gr/Si-M delivered superior cycling with 86.9% capacity retention and a low constant-voltage charging ratio of 19.6%, outperforming Gr/Si–S (77.4% and 20.6%) and bare Gr/Si (79.6% and 25.7%), confirming suppressed electrode resistance. As a typical breathing MOF, MIL-53(Al) reversibly switches pore sizes between 8.5 Å and 15.6 Å with over 40% volumetric variation. As stated in this literature, the flexible framework of MIL-53(Al) buffers silicon's volume variation and suppresses particle pulverization and detachment. Meanwhile, the material's intrinsic adhesion enables uniform silicon dispersion on graphite to mitigate localized stress concentrations. Its dynamically tunable pores further stabilize electrode interfaces and restrain excessive SEI growth. In contrast, rigid Super P lacks deformable pores to accommodate volume variation, leading to inferior performance. MIL-53, however, undergoes reversible deformation *via* its breathing effect, which mitigates silicon expansion while exhibiting high capacity retention.^69^

**Fig. 4:**
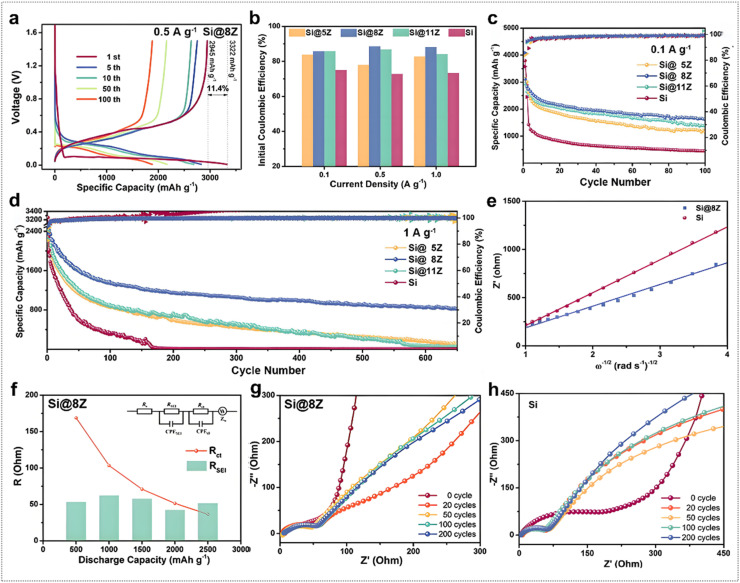
Electrochemical and impedance characterization of Si@ZIF-8 and pure Si.^76^ Adapted/reproduced from ref. 76 with permission from Elsevier, copyright 2023. (a) Si@8Z GCD curves at 0.5 A g^−1^. (b) Initial coulombic efficiencies; (c and d) long-cycle stability at 0.1/1 A g^−1^. (e) Low-frequency *Z*′ *vs. ω*^−1/2^ fitting. (f) Si@8Z resistance evolution. (g and h) Nyquist plots at various cycles.

As a typical gate-opening MOF, ZIF-8 realizes reversible pore aperture adjustment *via* organic linker swinging. Despite the absence of a reversible crystalline phase transition, it exhibits a high mechanical modulus of approximately 1.4 GPa.^35,70–72^ Upon compositing with silicon, core–shell Si@ZIF-8 is obtained with scattered rhombic dodecahedral ZIF-8 particles. Characterized by reversible gate-opening flexibility, ZIF-8 enables continuous pore aperture variation *via* reversible imidazolate linker rotation and works through multi-pronged effects: gate-derived elastic deformation buffers silicon's lithiation expansion to disperse stress and retain electrode integrity;^73–75^ hierarchical porous channels confine Si particles, realize Li^+^ sieving and desolvation, and accelerate ion transport for reduced charge-transfer resistance; additionally, surface Lewis-basic N sites induce formation of a uniform LiF-rich compact SEI, suppressing electrolyte decomposition and destructive SEI rupture.^73^ Section 4.2.2 details SEI film regulation mechanisms. The experiment confirms that the Si@ZIF-8 anode exhibits superior overall electrochemical performance to pure Si: as shown in Fig. 4a, the galvanostatic charge–discharge curves of the Si@8Z composite (92 wt% Si and 8 wt% ZIF-8) are presented. It retains a specific capacity of nearly 2000 mAh g^−1^ after 100 cycles at 0.5 A g^−1^; Si@ZIF-8 core–shell composites with ZIF-8 mass fractions of ∼5%, 8%, and 11% (corresponding to ∼95%, 92%, and 89% Si) are denoted as Si@5Z, Si@8Z, and Si@11Z, respectively. As shown in Fig. 4b, all Si@ZIF-8 samples exhibit significantly higher initial coulombic efficiency (ICE) than pure Si at 0.1, 0.5, and 1.0 A g^−1^, with Si@8Z (8 wt% ZIF-8) delivering the optimal performance, reaching a maximum ICE of ∼89% (*vs.* only 73.4% for pure Si). Fig. 4c and d present the long-cycle performance: Si@8Z retains a high specific capacity of 1614.5 mAh g^−1^ after 100 cycles at 0.1 A g^−1^, and retains a capacity above 800 mAh g^−1^ at 1 A g^−1^, whereas the capacity of pure Si decays to nearly zero. Electrochemical impedance tests (Fig. 4e–h) further verify that Si@8Z possesses a lower charge transfer resistance.

**Fig. 5:**
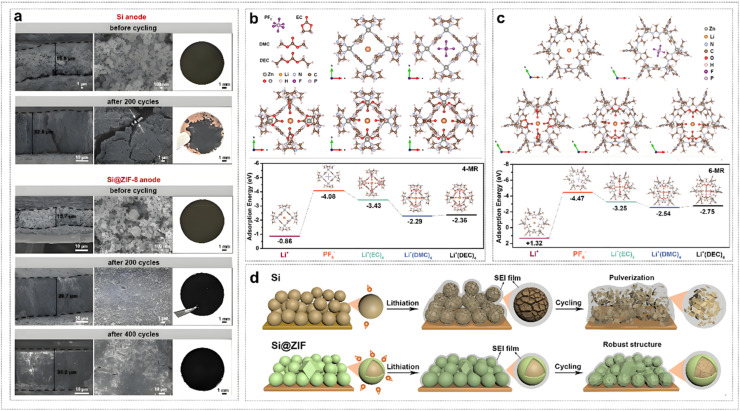
Morphology, calculations, and mechanism of Si and Si@ZIF-8 anodes.^76^ Adapted/reproduced from ref. 76 with permission from Elsevier, copyright 2023. (a) SEM/digital photos before/after cycling. (b and c) Adsorption energies of ZIF-8 4-/6-MR with Li species. (d) Schematic of cycling-induced structural evolution.

Moreover, Fig. 5a shows that after multiple cycles, Si@ZIF-8 has much better structural integrity than pure Si, with excellent structural stability and anti-volume expansion capability. Fig. 5b and c presents DFT adsorption energies of Li^+^, PF_6_^−^ and solvated lithium clusters on ZIF-8's 4-MR and 6-MR windows. PF_6_^−^ and solvated Li species show strongly negative adsorption energies and are trapped by pores; the 6-MR window repels bare Li^+^ with an adsorption energy of 1.32 eV, whereas the size-matched 4-MR channel enables rapid Li^+^ transport. Strong adsorption of solvent clusters enriches Li^+^ around pores, thereby boosting ionic diffusion and electrochemical kinetics. Consistent with previous reports, the results reveal reversible rotation of imidazolate linkers. ZIF-8 gate opening stems from continuous adsorption-triggered framework deformation that tunes sieving pore apertures, offering theoretical support for its inherent flexible gate-opening feature.^77^ Meanwhile, Fig. 5d shows that after cycling, the Si@ZIF-8 electrode has a LiF-rich, intact SEI membrane and stable ZIF-8 framework.^76^ In summary, the catalytic, reversible structural and interfacial regulation of these MOFs mitigates silicon's volume expansion and sustains stable high capacity.

Gate-opening flexible ZIF-67 is used to modify silicon anodes, where rotatable 2-methylimidazole ligands realize tunable pore channels. Considering the severe volume expansion of silicon during Li^+^ insertion/extraction, the composite is expected to dynamically accommodate silicon volume variation upon cycling and retain the intact ZIF framework structure.^78–80^ Pristine ZIF-67 without calcination was used to synthesize ZIF-67 coated silicon nanoparticles (Si@ZIF) *via* a one-pot hydrothermal method. Combined with papermaking technology, cellulose-based flexible free-standing electrodes were prepared without PVDF binders and toxic NMP. ZIF-67 relieves silicon volume expansion and electrode failure *via* physical confinement. Its hierarchical pores accelerate lithium-ion transport and electrolyte infiltration and regulate lithium solvation to stabilize the SEI film. Si@ZIF has a specific surface area of 1306.15 m^2^ g^−1^ and a silicon loading of 30.39 wt%, outperforming carbonized MOFs in terms of pore characteristics. Electrochemical evaluations confirm outstanding cycling stability: the half-cell exhibits 1744.6 mAh g^−1^ after 1000 cycles at 0.5 A g^−1^. The LFP/Si@ZIF full cell retains 80.5% capacity over 3400 cycles, and the flexible pouch cell delivers 85.2% capacity retention and nearly 99.6% coulombic efficiency after 500 cycles. The enhanced performance is attributed to the dual buffering structure, accelerated ion kinetics, optimized interfacial environment and synergistic conductive network. Moreover, this scalable preparation method enables low-cost and biodegradable electrodes.^81^ The stress-buffering mechanisms employed in this material are consistent with those observed in analogous systems. Performance variations primarily stem from differences in composite formulation and structural architecture.

**Fig. 6:**
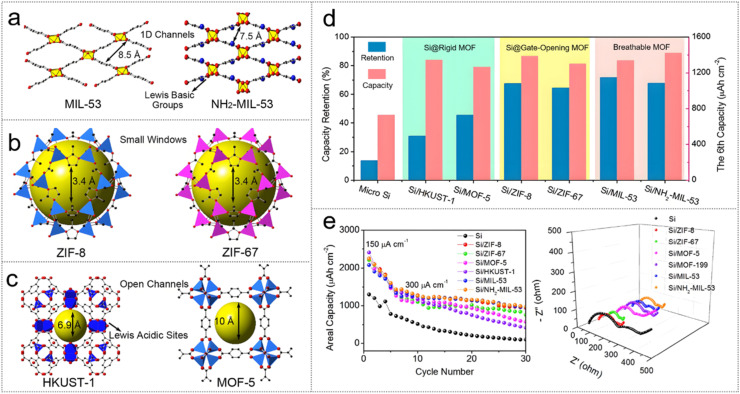
MOF structures and electrochemical behaviors of Si@MOF anodes.^53^ Adapted/reproduced from ref. 53 with permission from American Chemical Society, copyright 2015. (a) 1D channels of flexible MIL-53/NH_2_-MIL-53 with Lewis basic groups. (b) Gate-opening ZIF-8/ZIF-67 cages with 3.4 Å windows. (c) Rigid HKUST-1/MOF-5 with labeled pores and Lewis acid sites. (d) Capacities of the sixth cycle and capacity retentions of micro-Si and micro-Si coated with different MOFs after 30 cycles. Si/MOF represents the structure with the surface coated with different MOFs. (e) Left: areal capacity cycling at 150/300 µA cm^−2^; right: 3D EIS Nyquist spectra.

Another study proposed the MOF interlayer coating strategy (MOF-SC) to fabricate layered electrodes with a structure of copper foil/Super P/silicon/MOF coating. Serving as a functional buffer layer, MOF was applied to modify silicon anodes for lithium-ion batteries, aiming to resolve silicon's inherent problems including severe volume expansion, electrode pulverization and short cycle life during Li^+^ intercalation and deintercalation. The research used both micrometer and nanometer silicon to evaluate six typical MOFs, which were divided into three groups according to their framework dynamic characteristics. Breathable MOFs (MIL-53 and NH_2_-MIL-53) have one-dimensional dynamic channels and distinct breathing behavior; their structure is shown in Fig. 6a. Their frameworks and pores expand and contract reversibly upon cycling. Amino side groups only modify pore surfaces without changing the original breathing properties. Gated MOFs (ZIF-8 and ZIF-67) belong to zeolitic imidazolate frameworks. Their structure is shown in Fig. 6b. Their organic linkers swing to regulate pore apertures, and the flexible skeleton can mildly accommodate volume changes. Rigid MOFs (MOF-5 and HKUST-1/Cu-MOF-199) feature fully fixed frameworks and large open pores. Their structure is shown in Fig. 6c. Although HKUST-1 contains Lewis acid sites, rigid MOFs can only exert passive physical confinement *via* inherent pores. Targeted electrochemical characterization was performed on C/Si/ZIF-8 composite electrodes derived from gate-opening ZIF-8. At a current density of 265 µA cm^−2^, the micrometer silicon-based electrode delivered an initial areal capacity of 1700 µAh cm^−2^ and retained 850 µAh cm^−2^ after 50 cycles. Post-cycle PXRD and SEM characterization confirmed that ZIF-8 retained an intact crystal structure and coating morphology without detachment or collapse. When cycled at 425 µA cm^−2^, the nanometer silicon-based C/Si/ZIF-8 electrode exhibited a stable areal capacity of 600 µAh cm^−2^ over 100 cycles with no obvious fading. Taking 30-cycle capacity retention as the evaluation index, Fig. 6d compares the 6th-cycle capacity and 30-cycle retention of bare micro-Si and various MOF-coated micro-Si, where Si/MOF refers to MOF-encapsulated silicon composites. The six MOFs show performance ranking as follows: MIL-53 (72%), NH_2_-MIL-53 (68%), ZIF-8 & ZIF-67 (65%), MOF-5 (45%) and HKUST-1 (31%). Fig. 6e displays 30-cycle data of bare micro-Si and Si/MOF electrodes (150 µA cm^−2^ for the first 5 cycles and 300 µA cm^−2^ for the rest). All MOF-coated electrodes deliver higher initial areal capacity and better cycling stability, with performance ranked as NH_2_-MIL-53 > MIL-53 > ZIF-67 > ZIF-8 > rigid HKUST-1/MOF-5. EIS Nyquist spectra confirm smaller charge-transfer impedance of Si/MOF samples, whose impedance trend aligns with their cycling performance.^53^ The results prove that framework flexibility and dynamic pores effectively enhance the cycling stability of silicon anodes, and breathable/gated MOFs exhibit better modification performance than large-pore rigid MOFs. Mechanistically, MIL-series breathable MOFs realize adaptive elastic buffering through reversible breathing behavior. Their dynamically adjustable pores perfectly match silicon's volume fluctuation, accounting for their optimal modification effect. ZIF-series gated MOFs achieve gradual pore regulation *via* reversible linker swinging and provide moderate elastic buffering. By contrast, rigid MOFs such as MOF-5 and HKUST-1 have static frameworks. They rely merely on porous structures for physical confinement and cannot adapt to silicon's drastic deformation. The weak confinement eventually causes rapid capacity decay.^53,67,82^

Overall, breathable MOFs undergo reversible deformation to dynamically adjust pores and accommodate silicon's cyclic volume variation during cycling, while gate-opening MOFs buffer stress *via* passive pore regulation. Both approaches address the trade-off between high capacity and volume expansion, breaking the inherent contradiction between capacity and structural stability of silicon anodes. For practical applications, however, they still have evident limitations. Breathable MOFs show environmentally sensitive dynamic behavior and low intrinsic conductivity.^83,84^ Gate-opening MOFs possess a lower framework mechanical modulus, making them vulnerable to microstructural damage under high-load conditions.^85^ In addition, Si@MOF composites can be synthesized *via* numerous routes with various silicon precursors. Though breathable/gate-opening MOFs excel in stress buffering and electrochemical capacity, practical material selection and composite design require a full trade-off among cost, performance and environmental benefits.

#### 4.1.2 Rigid MOFs

Rigid MOFs are assembled from high-coordination metal clusters and rigid aromatic ligands *via* robust coordination bonds. Their framework and pore size remain invariant during guest sorption and Li^+^ cycling, free from phase transition or gate-opening breathing, producing Langmuir isotherms unlike the S-shaped curves of breathable MOFs.^45^ Featuring outstanding thermal, chemical and mechanical stability but no stimulus-responsive deformation, they encapsulate silicon to passively relieve volume expansion through intrinsic pores; unsaturated metal sites optimize interfacial properties, suppress side reactions and guarantee favorable conductivity. Nevertheless, unidirectional spatial confinement limits capacity output and makes the framework vulnerable to stress fracture under long-term cycling.^44^

**Fig. 7:**
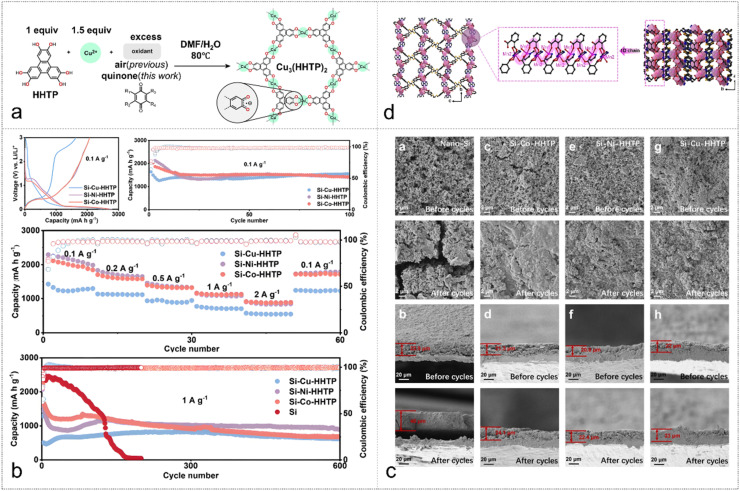
(a) Synthesis of Cu_3_(HHTP)_2_: the HHTP ligand assembles with Cu^2+^ in a DMF/water mixture at 80 °C under oxidation, with key intermediates shown in the figure.^87^ Adapted/reproduced from ref. 87 with permission from Royal Society of Chemistry, copyright 2022. (b) The electrochemical test performance of Si-M-HHTP. (c)Surface and cross-sectional SEM images of nanoSi, Si–Co-HHTP, Si–Ni-HHTP, and Si–Cu-HHTP before and after 100 cycles at 1 A g^−1^.^92^ Adapted/reproduced from ref. 92 with permission from American Chemical Society, copyright 2025. (d) Mn-*o*-TTFOB structure: 3D views along the *bc* and *ab* planes and its 1D chain structure.

Unlike flexible MOFs such as ZIF-8 that feature gate-opening and breathing effects, M-HHTP (M = Co, Ni, Cu) is a rigid conductive MOF. As shown in Fig. 7a, Cu^2+^ coordinates with oxygen atoms of HHTP ligands, and d–π conjugation between metal 3d orbitals and ligand π electron clouds forms continuous in-plane electron transport pathways.^86–88^ Built from 2D rod-like units, M-HHTP constructs a stable 3D rigid network, which undergoes no ligand swing, crystal phase transition or reversible framework deformation during lithiation and delithiation. As reported by Jun Chen's group, M_3_(HHTP) exhibits outstanding intrinsic conductivity. In particular, Cu_3_(HHTP) has a conductivity of 2.1 × 10^−1^ S cm^−1^, far surpassing that of conventional MOFs (<10^−10^ S cm^−1^).^86^ Si-M-HHTP composites are synthesized *via* a one-pot method. The rigid conductive M-HHTP improves nanoSi anodes through multiple synergistic effects. For electron transport, the extensive d–π conjugation system and tight interfacial contact between the 3D framework and silicon greatly reduce interfacial impedance. EIS measurements reveal that the charge transfer resistance of Si–Co-HHTP, Si–Ni-HHTP and Si–Cu-HHTP is 52.63 Ω, 31.23 Ω and 49.53 Ω, respectively, all much lower than 583.2 Ω of pure nano-Si; Si–Ni-HHTP presents the optimal charge transport performance. In terms of ion conduction, the porous rigid framework and reversible electrochemical interactions between metal active sites and Li^+^ jointly accelerate Li^+^ migration. Structurally, the 3D network acts as physical confinement to buffer silicon's ∼300% volume expansion.^89–91^ Meanwhile, HF treatment reduces the silicon particle size from 100 nm to 50 nm for further volume suppression. Robust metal–oxygen bonds guarantee long-term structural integrity of M–HHTP, and the varied redox properties of different metal ions lead to performance discrepancies among composites. Electrochemical results in Fig. 7b show that all Si–M-HHTP samples deliver an initial discharge capacity above 2000 mAh g^−1^ at 0.1 A g^−1^, with nearly 100% coulombic efficiency and over 90% capacity retention after 100 cycles. After 600 cycles at 1 A g^−1^, Si–Ni-HHTP still retains a reversible capacity of 891.4 mAh g^−1^, and all composites achieve a capacity recovery rate above 90% in rate tests. SEM images (Fig. 7c) confirm the structural protection effect: after 100 cycles at 1 A g^−1^, pure silicon electrodes suffer severe cracking and thickness expansion from 22.5 µm to 40 µm, accompanied by active material detachment from the current collector. In contrast, Si-M-HHTP electrodes remain nearly intact. Overall, the rigid conductive M-HHTP addresses silicon's two major drawbacks, severe volume expansion and sluggish charge transport, *via* stress relief, physical confinement and electrochemical optimization.^92^ Three isomorphic 3D conductive MOFs (M_4_(*o*-TTFOB)(bpm)_2_(H_2_O)_2_ (where M is Mn, Zn, and Cd; o-H8TTFOB is *ortho*-tetrathiafulvalene octabenzoate; and bpm is 2,2-bipyrimidine)) developed by Zuo Jinglin's group are typical rigid frameworks. Their structure is shown in Fig. 7d. Each *o*-TTFOB^8−^ ligand coordinates with ten metal ions to form highly connected networks, which endow the materials with superior structural stability: they remain intact across pH 3–11 and in various organic solvents, with a thermal decomposition temperature of 320 °C. These MOFs exhibit excellent lithium storage performance. The Zn-based sample delivers a high reversible capacity of 1288 mAh g^−1^, and all samples show outstanding rate capability and cycling stability with nearly 100% coulombic efficiency. Intermolecular S⋯S interactions within TTF moieties and the 3D coordination network jointly enhance electron transport, achieving a room-temperature conductivity of 10^−7^–10^−8^ S m^−1^. Li^+^ preferentially adsorbs on ligand oxygen sites, with a negative adsorption energy ranging from −2.36 to −2.13 eV, and *ortho*-carboxyl groups reduce the Li^+^ intercalation free energy. Lattice amorphization during cycling exposes abundant active sites, raising the capacitive contribution to 42–58%. Benefiting from the 3D porous structure, the Li^+^ conductivity and Li^+^ diffusion coefficient reaches the order of 10^−10^ cm^2^ s^−1^. Combining rigid skeletons, high conductivity and fast ion transport, this type of MOF holds great potential for integration with silicon for high-performance anode fabrication. Zhang Qichun's group synthesized a butterfly-shaped π–d conjugated Cu-DBC MOF. This material is a rigid conductive framework and can serve as an anode for lithium-ion batteries. Its core mechanism lies in strong π–d conjugation (band gap narrowed to 0.303 eV) between Cu^2+^ d-orbitals and ligand π-electron clouds, synergized with dual redox (Cu^2+^/Cu^+^ and [Cu(OR)_4_]^3−^/[Cu(OR)_4_]^2−^) and diffusion–capacitance dual storage mechanisms. Supported by a rigid framework and strong coordination bonds for stability, it achieves a 2500-cycle long lifespan.^91^ Rigid MOFs like Cu-DBC are rarely explored for modifying silicon anodes, but their rigid mesoporous architecture, π–d conjugation and dual lithium storage enable them to mitigate volume expansion, improve charge/ion transport and stabilize SEI films, showing great application potential.

Rigid MOFs span a wide range of structures, and their electrochemical cycling performance (represented by MOF-5 and HKUST-1/Cu-MOF-199) has been compared with breathable/gate-opening MOFs in the preceding section.^53^

Overall, rigid MOFs deliver outstanding thermal, chemical and mechanical stability. Their intrinsic rigid porous frameworks physically confine and buffer the dramatic volume expansion of silicon materials. Meanwhile, unsaturated metal active sites optimize electrode interfacial environments, suppress detrimental side reactions and stabilize SEI films. Nevertheless, rigid MOFs have inherent drawbacks for silicon anode modification: unidirectional spatial confinement hinders full capacity release, and rigid skeletons easily suffer stress-induced cracking from volumetric fluctuations during long-term cycling.^53^ Furthermore, high-performance conductive rigid MOFs such as Cu-DBC are barely applied to silicon anode modification, yet they possess enormous developmental potential.

#### 4.1.3 Derivatives of MOFs

At 600–1000 °C under an inert atmosphere, rigid and breathable MOFs transform into N/O/S heteroatom-doped porous carbon composites anchored with metal/metal oxide nanoparticles, effectively resolving the volume expansion and low conductivity issues of silicon-based anodes. High temperature breaks the majority of MOF coordination bonds, triggering different structural changes: breathable MOFs can be synthesized under an inert atmosphere to obtain metal/metal oxide doped MOF-derived carbons with their original morphology and high specific surface area. However, their breathability is lost whereas rigid MOFs densify into structurally stable carbon frameworks. The *in situ* heteroatom doping forms continuous conductive networks with tunable conductivity of 10^2^–10^3^ S m^−1^ to boost electron transport, and the hierarchical porous structure of carbon frameworks efficiently buffers silicon volume expansion. Uniformly dispersed metal/metal oxide nanoparticles accelerate reaction kinetics and Li^+^ migration. Specifically, N doping induces electrostatic interaction with surface SiO_*x*_, promoting electron transfer and suppressing particle agglomeration. Furthermore, the outer carbon shell separates silicon from electrolyte, stabilizes SEI films and reduces dead silicon generation, synergistically improving the electrochemical performance of silicon anodes.^38,93–95^

**Fig. 8:**
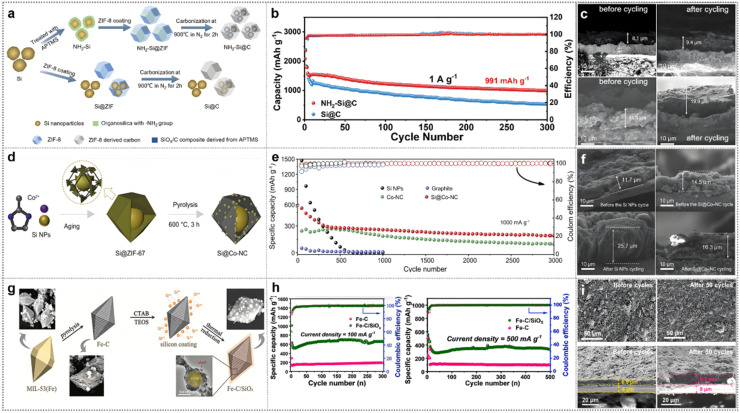
Fabrication, performance and stability of three MOF-derived carbon-coated Si anodes. (a–c) ZIF-8-based Si@C anodes: fabrication, cycling performance and pre/post-cycling SEM.^96^ Adapted/reproduced from ref. 96 with permission from Elsevier, copyright 2025. (d–f) ZIF-67-based Si@Co-NC anodes: fabrication, long-cycle performance and pre/post-cycling SEM.^42^ Adapted/reproduced from ref. 42 with permission from KeAi Publishing Communications Ltd, copyright 2022. (g–i) MIL-53(Fe)-based Fe–C/SiO_*x*_@Si anodes: fabrication, cycling performance and pre/post-cycling characterization.^97^ Adapted/reproduced from ref. 97 with permission from Elsevier, copyright 2025.

There have been many studies on ZIF calcination. In typical cases, APTMS-surface-aminated Si nanoparticles self-assemble with Zn^2+^ and 2-MIM into NH_2_–Si@ZIF, which is then pyrolyzed at 900 °C in N_2_ for 2 h to form ZIF-8-derived N-doped carbon matrix-encapsulated Si (NH_2_–Si@C). Amination enhances Si-ZIF-8 coordination, preserving post-pyrolysis encapsulation integrity and enabling *in situ* N-doping (Fig. 8a). Fig. 8c shows the SEM images before and after the cycle, allowing for observation of the volume changes. Pyrolysis-formed porous carbon suppresses Si volume expansion to 14.3% and lowers charge transfer impedance for better conductivity, with N-doping strengthening carbon–Si interactions. It exhibits superior electrochemical performance: 991 mAh g^−1^ (66.3% retention) after 300 cycles at 1 A g^−1^ (Fig. 8b), 1062 mAh g^−1^ at 5 A g^−1^, and a Li^+^ diffusion coefficient of 10^−11^ cm^2^ s^−1^.^96^

Another study reported that Si nanoparticles combined with ZIF-67 undergo thermal decomposition at 600 °C in N_2_ for 3 hours, forming the hollow core–shell Si@Co-NC composite with Si confined in carbon shell voids (Fig. 8d). Thermal decomposition enables co-doping of N (pyridine, pyrrole, and graphite N) and Co particles; N doping enhances carbon matrix defects, optimizes charge distribution, and lowers work function, synergizing with Co to boost electron conductivity and Li^+^ diffusion.^98^ The carbon shell and voids suppress Si volume expansion. Fig. 8f shows cross-sectional SEM comparison of Si nanoparticles and Si@Co-NC before and after cycling. Si nanoparticles exhibit ∼120% volume expansion (11.7 µm to 25.7 µm) with severe structural collapse, while Si@Co-NC only shows ∼12.4% expansion (14.5 µm to 16.3 µm) with an intact structure, confirming that the Co-NC coating effectively suppresses Si volume expansion and stabilizes the electrode structure, and the material exhibits excellent electrochemical performance: 191.6/191.4 mAh g^−1^ at 1000 mA g^−1^ over 3000 cycles (Fig. 8e), 100% coulombic efficiency, 0.022% per cycle capacity loss, 156.9 Ω charge transfer impedance, and stable high-rate capacity, far outperforming pure Si and commercial graphite anodes.^42^

Studies have shown that breathable MIL-53(Fe) was vacuum-calcined at 800 °C to rupture dynamic coordination bonds and destroy its native breathing framework, producing rigid Fe–C porous carbon embedded with Fe/Fe_3_O_4_ nanoparticles. Core–shell Fe–C/SiO_*x*_ anodes were then synthesized *via* a CTAB-guided TEOS hydrolysis coating followed by secondary annealing. The process and structure are shown in Fig. 8g. As shown in Fig. 8h, the composite delivers an initial discharge capacity of 658 mAh g^−1^ with an initial coulombic efficiency of 60% at 0.1 A g^−1^, retaining 650 mAh g^−1^ after 300 cycles. At 0.5 A g^−1^, its reversible capacity remains above 300 mAh g^−1^ over 500 cycles, demonstrating outstanding cycling and rate performance. High-temperature pyrolysis irreversibly removes the ligand-triggered reversible flexibility of pristine MIL-53(Fe); the as-obtained rigid carbon only passively accommodates SiO_*x*_ expansion *via* intrinsic mesopores, leading to a 34.8% electrode volume rise after 50 cycles. The specific SEM image is shown in Fig. 8i. While such swelling is far restrained relative to bare bulk SiO_*x*_ and silicon, the rigid skeleton cannot synchronously expand or contract to dissipate cyclic stress. Benefiting from the continuous conductive network constructed by carbon and iron nanoparticles, interfacial impedance is reduced, SEI film evolution is optimized and dead silicon formation is inhibited. Accordingly, MOF-derived rigid carbon surpasses pristine SiO_*x*_ in cycling stability, yet underperforms electrodes modified with as-synthesized, uncalcined breathable MIL-53(Al) on account of lost structural adaptivity.^69,97^

ZIF-series MOFs are widely coupled with silicon *via* calcination to fabricate carbon-encapsulated silicon electrodes. One route involves direct coating of silicon with crystalline ZIF-8 (denoted Si@ZIF-8) through a room-temperature one-pot method, while the other adopts high-temperature ZIF-8 pyrolysis followed by *in situ* silicon growth, differing greatly in silicon raw materials and composite assembly logic.

The preceding section has covered ZIF-8/Si composite anodes; herein, we detail two distinct fabrication strategies and their characteristic structural configurations. For the pyrolysis-derived hybrid: ZIF-8 is carbonized at 900 °C under argon to obtain porous carbon embedded with zinc active sites. After acid washing to eliminate surface impurities, 2–3 nm silicon nanodots are *in situ* grown within carbon channels at 540 °C under negative pressure, with silane as the silicon source and residual zinc as the catalyst. This ZIF-8 carbon/silicon composite exhibits negligible volume strain and superior long-cycle stability. At 1 A g^−1^, it retains a reversible capacity of 1172 mAh g^−1^ after 1000 cycles, accompanied by merely 3% overall electrode expansion and an initial coulombic efficiency (ICE) of 87.94%. The remarkable performance arises from the synergistic effect of porous carbon frameworks: spatial confinement restrains silicon volume fluctuation, rigid carbon matrices release internal stress during lithiation/delithiation, interconnected conductive networks accelerate charge transport, and residual zinc sites modulate electrode interfacial properties.^99^ By contrast, the room-temperature synthesized Si@ZIF-8 supports kilogram-scale mass production and inherits the intrinsic gate-opening flexible structure of crystalline ZIF-8. It delivers an ICE of 88.2% and outstanding ultrahigh-rate performance up to 8 A g^−1^, yet suffers inferior cycling durability, only retaining 818.5 mAh g^−1^ after 650 cycles at 1 A g^−1^.

Though both composites employ ZIF-8 as the precursor, their performance disparities originate from silicon particle size, matrix structure and working mechanisms. The pyrolytic composite takes full advantage of ultrafine 2–3 nm silicon nanodots to drastically mitigate volumetric deformation, and its conductive carbon skeleton together with tight interfacial bonding further boosts cycling lifespan. For Si@ZIF-8 loaded with large silicon particles, the elastic gate-opening effect of crystalline ZIF-8 serves as an effective volume buffer. In summary, pyrolysis-derived ZIF-8 carbon/silicon materials are more suitable for lab-based high-performance research, whereas crystalline ZIF-8 coated silicon anodes feature simple preparation and reliable gate-opening buffering capacity, demonstrating greater commercialization potential. The two modification strategies respectively realize carbon matrix reconstruction and utilize the inherent flexibility of MOFs to meet diverse application demands.^76,99^

**Fig. 9:**
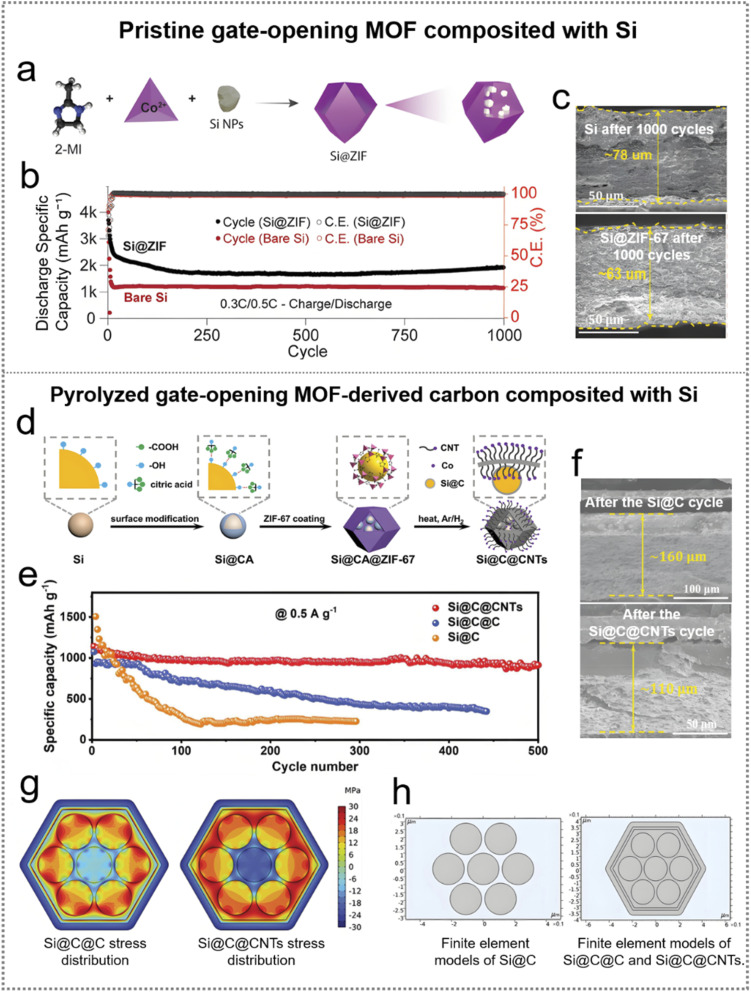
Comparative characterization of the two ZIF-67-modified silicon anodes: the pristine unpyrolyzed gate-opening ZIF-67/Si composite and pyrolyzed ZIF-67-derived carbon/Si composite.^81,93^ Adapted/reproduced from ref. 81 with permission from Wiley-VCH Verlag, copyright 2024. Top: (a) synthesis of Si@ZIF, (b) long-cycle performance, and (c) post-cycling electrode SEM. Bottom: (d) fabrication of Si@C@CNTs, (e) cycling curves, (f) cycled electrode morphology, and (g and h) finite element stress simulation. Adapted/reproduced from ref. 93 with permission from Wiley-VCH Verlag, copyright 2023.

ZIF-67 has been widely exploited in lithium-ion battery anodes, yet Si@ZIF-67 composites reported across the literature deliver divergent electrochemical performance, which is determined by multiple factors including intrinsic gate-opening behavior, calcination treatment, composite strategies and structural design.^93^ As previously discussed, the gate-opening MOF in this work is pristine ZIF-67 without pyrolysis, and its fabrication route is presented in Fig. 9a. A cellulose-based free-standing flexible electrode was also fabricated using this composite. For comparison, we further analyze ZIF-67 derivatives treated by high-temperature pyrolysis. These pyrolyzed carbon-based composites are designed to optimize mechanical stress relief and electrical conductivity. Their dual-carbon/carbon nanotube (CNT) three-dimensional network homogenizes lithiation-induced stress and inhibits electrode cracking, which is fully supported by the finite element stress distribution models of Si@C, Si@C and Si@CNTs shown in Fig. 9g and h. Cobalt nanoparticles work synergistically with CNTs to construct continuous conductive pathways, while the porous carbon matrix shortens lithium-ion diffusion lengths. The synthetic procedure of the pyrolyzed composite is illustrated in Fig. 9d. At a current density of 0.5 A g^−1^, the material delivers a reversible capacity of nearly 1000 mAh g^−1^ after 500 cycles (Fig. 9e). It exhibits excellent high-rate stability but a relatively low specific capacity overall. Cross-sectional SEM results (Fig. 9f) confirm that high-temperature treatment effectively alleviates the volume swelling of silicon electrodes after cycling.

In contrast, the pristine flexible gate-opening ZIF-67 functions *via* framework confinement, ion regulation and interfacial engineering. Its flexible framework can adapt to silicon's volume deformation and prevent particle pulverization. Fig. 9c demonstrates its superior capability in suppressing electrode expansion compared with that of pyrolyzed samples. The hierarchical pore structure improves electrolyte infiltration, and the pore confinement effect optimizes lithium-ion solvation environments and stabilizes the SEI film. The Si@ZIF half-cell retains a reversible capacity of 1744.6 mAh g^−1^ at 0.5 A g^−1^ (Fig. 9b). When paired with LFP as the cathode, the full cell retains 80.5% of its initial capacity after 3400 cycles. The flexible pouch cell achieves 85.2% capacity retention and a stable coulombic efficiency of 99.6% over 500 cycles. Evidently, pristine ZIF-67 possesses prominent advantages in reversible capacity and long-term cycling performance.^81,93^

Another study employed two rigid and inherently insulating MOFs, Co-MOF-74 and Cu-MOF-199 (HKUST-1). Both feature stable 3D porous architectures without framework breathing or gate-opening effects and were employed to modify silicon anodes. A series of comparative electrodes were prepared: pure silicon and silicon–graphite electrodes served as controls; uncalcined samples included simple Si-MOF blends and Super P/silicon/MOF sandwich electrodes. For calcined samples, Co-MOF-74 was grown *in situ* on silicon nanoparticles and pyrolyzed at 800 °C under argon, yielding low-silicon 0.5Si@MOF-c and high-silicon 1.0Si@MOF-c. Electrochemical tests showed that pristine MOFs suffer from poor conductivity, which undermines the performance of uncalcined electrodes. The simple Si-MOF blends exhibited sluggish charge transport and fluctuating coulombic efficiency, retaining only 25% capacity after 30 cycles. The sandwich electrodes partially compensated for conductivity drawbacks *via* Super P, achieving a capacity retention of 40–55% after 100 cycles; those based on Cu-MOF-199 decayed faster and developed obvious cracks after cycling. By comparison, pure silicon electrodes retained merely 21% capacity over 100 cycles, and silicon–graphite electrodes held around 40%. After pyrolysis, MOF precursors were converted into porous carbon matrices that resolved the insulation issue and effectively confined silicon particles. The low-silicon 0.5Si@MOF-c delivered an initial reversible capacity of ∼1000 mAh g^−1^ with a 100-cycle retention of 60%, while the high-silicon 1.0Si@MOF-c showed an initial capacity of 1900 mAh g^−1^ but only 25% retention after 100 cycles due to silicon agglomeration and excessive SEI growth. Mechanistically, the rigid MOF skeletons disperse mechanical stress during silicon (de)lithiation, suppressing particle pulverization and electrode delamination; the sandwich structure relieves bulk deformation, and *in situ* encapsulation restricts silicon at the microscale. MOF and carbon coatings isolate silicon from electrolytes to stabilize the SEI film, whereas the high impedance of uncalcined MOFs causes uneven charge distribution and aggravated side reactions. Without continuous conductive pathways, uncalcined electrodes suffer from severe polarization and rapid capacity fading, while pyrolytic porous carbon builds integrated conductive networks to lower charge transfer resistance and accelerate lithium-ion diffusion. In summary, the rigid confinement of pristine MOFs can mitigate silicon's inherent defects, yet their low conductivity limits the overall improvement. Pyrolysis-carbonization preserves the structural confinement while greatly boosting conductivity, leading to superior electrochemical performance. The sandwich design also offers a new strategy to balance capacity and volume expansion, and multi-component synergy is expected to further optimize electrode performance in future research.^100^

Another study further validates the excellent applicability of rigid porous In-MOF. Adopting this rigid-structured In-MOF as the precursor, In_2_O_3_/C hybrids can be obtained *via* annealing treatment, which are further *in situ* coated on nanosilicon to construct high-performance Si@In_2_O_3_/C composite anodes. The Li–In alloy (low Young's modulus for stress buffering) and amorphous Li_2_O formed during In_2_O_3_ lithiation enhanced ionic conductivity; the porous carbon in the composite provided electron transport channels and volume buffer space, synergistically inhibiting Si pulverization and agglomeration. At a high loading of 7.64 mg cm^−2^ and a current density of 2.74 mA cm^−2^, the anode exhibited a specific capacity of 1093.2 mAh g^−1^ with an 84.3% retention after 500 cycles (only 5.5% for pure Si). The full cell assembled with LNO@NCM achieved a 74.3% capacity retention and a 99.94% average coulombic efficiency after 1000 cycles at 0.2C, with significantly reduced charge transfer impedance and volume expansion.^101^

Carbon derivatives from high-temperature pyrolysis of rigid, breathable and gated MOFs serve as promising modifiers for silicon anodes. Thermal treatment converts MOF precursors into robust rigid porous frameworks and eliminates the reversible breathing feature of breathable MOFs. The rigid skeleton confines silicon nanoparticles, buffers cyclic volume change, suppresses particle pulverization and interfacial side reactions, and stabilizes the SEI film. Meanwhile, *in situ* carbon conductive networks and metal/metal oxide active sites improve electrode conductivity, lower charge transfer impedance and accelerate lithium-ion transport, enhancing the cycling stability and high-loading performance of silicon anodes. These rigid structures, however, have no structural reversibility or adaptive breathing ability. They rely on fixed pores to passively accommodate silicon volume expansion, resulting in capacity loss; long-term cyclic mechanical stress also tends to fracture rigid carbon frameworks. Compared with pristine flexible breathable MOFs, MOF-derived rigid composites cannot balance volume buffering and capacity retention, limiting their application in long-cycle and high-energy-density batteries. Despite this drawback, their excellent conductivity enhancement is irreplaceable. Accordingly, high-performance silicon anodes demand comprehensive optimization *via* trade-offs among expansion suppression, capacity retention, active site regulation and microstructure engineering. Table 2 shows the electrochemical performance of different types of MOFs and MOFs processed by different methods after being combined with silicon.

**Table 2:** Electrochemical performance and volume expansion suppression of Si anodes modified with diverse MOFs and MOF-derived carbon^a^

Electrode	MOF	Initial DC/CC (mAh g^−1^)	ICE (%)	CN	RC/rate (mA g^−1^) or capacity retention	Volume expansion rate	Ref.
(Graphite/Si-MIL-53)	MIL-53(Al)	—	96.5	100	86.9%	—	69
Si@8Z(8%ZIF-8)	ZIF-8	3322.8/2945.2	88.6	100	1614.5 (0.5 A g^−1^)	89.1%	76
Si@ZIF-67	ZIF-67	6537.8/4725.4	72.3	1000	1744.6 (0.5 A g^−1^)	15%	81
C/Si/ZIF-8	ZIF-8	2046/1752	85.6	50	850 (265 µA cm^−2^)	—	53
Si/MIL-53	MIL-53	—	—	30	72%	—	53
Si–Co-HHTP	Co-HHTP	2689.4/2074.9	77.15	100	1434.6 (0.1 A g^−1^)	13.1%	92
Si–Ni-HHTP	Ni-HHTP	2785.9/2073.0	74.41	100	1405.7 (0.1 A g^−1^)	7.6%	92
Si–Cu-HHTP	Cu-HHTP	2541.5/1639.0	64.49	100	1553.4 (0.1 A g^−1^)	4.5%	92
NH_2_–Si@C	ZIF-8 derived N-doped C	2408/1825.3	100	300	1494 (1 A g^−1^)	14.3%	96
Si@Co-NC	ZIF-67 derived C	1098.9/797.1	72.5	80	565.8 (100 mA g−1)	12.4%	42
Fe–C/SiO_*x*_	Carbonized MIL-53(Fe)@SiO_*x*_	1013/608	60.0	300	650 (100 mA g−1)	34.8%	97
Si NDs ⊂ MDN	ZIF-8-derived MDN carbon	1593/1400	87.94	1000	1172 (1 A g^−1^)	3%	99
Si@C@CNTs	ZIF-67 derived C-CNT	1607/unmarked	—	500	968 (0.5 A g^−1^)	47%	93
10SiZGC (10 wt% Si@ZIF-62 glass)	ZIF-62 glass	1010/627	62.0	500	650 (1 A g^−1^)	—	37

a DC/CC: discharge capacity/charge capacity; ICE: initial coulombic efficiency; CN: cycle number; RC: reversible capacity.

### 4.2 Mechanism of MOF-based modification on silicon anodes

#### 4.2.1 The rationale behind the MOF-based scaffold-mediated capacity–volume trade-off

MOF electrodes can mediate the trade-off between specific capacity and volume variation through four universal structural strategies: flexible breathing frameworks, gated pore channels, rigid locked coordination networks, and MOF-derived carbon confinement. The corresponding atomic-scale mechanisms and supporting experimental evidence are summarized as follows.^44^

The coordination bonds linking metal nodes and organic linkers possess flexible degrees of freedom, allowing organic linkers to rotate, bend and slide. Interpenetrated stacking frameworks further deliver structural deformability. Upon external stimuli, reversible displacement of internal framework atoms and linkers occurs to trigger overall reversible structural phase transitions between narrow-pore and large-pore states. Such framework deformation is synergistically governed by dynamic coordination bond variation, conformational alteration of linkers and weak intermolecular interactions.^26^

For MIL-53(Al), benefiting from its intrinsic adhesive properties, it achieves homogeneous dispersion of silicon nanoparticles to mitigate localized stress concentration. Its flexible porous framework with reversible deformability accommodates the volumetric fluctuation of silicon and maintains structural integrity of electrodes, while the tunable pore channels facilitate lithium-ion transport and stabilize electrode interphases. By contrast, rigid conductive additives are devoid of deformable cavities for mechanical stress dissipation, thus delivering inferior long-term cycling durability.^69^ From a mechanistic perspective, the reversible wine-rack-type framework breathing of MIL-53 originates from atomic-scale bending of BDC carboxylate coordination hinges. Normalized pore width parameter *b*′, pore angle *Ψ*, and unit cell volume *V*_C_ exhibit linear correlations, verifying continuous and gradual structural deformation rather than a discrete two-phase transition. Pore-confined water molecules lock the framework into closed-pore conformations *via* hydrogen bonding. For Cr-based MIL-53, the *I*2/*a* monohydrate phase features a water occupancy of 0.43 with a unit cell volume of 975.0 Å^3^, while the fully hydrated *P*2_1_/*c* phase reaches 1985.2 Å^3^. A hemihydrate intermediate with a water occupancy of 0.25 and a unit cell volume of 1128.8 Å^3^ is additionally identified in Ga analogues, confirming that higher water content induces more severe framework contraction. At ambient pressure, the high desorption barrier of water restricts Cr-MIL-53 to only two isolated phases: an anhydrous closed-pore phase formed at 348 K and a fully open-pore phase generated at 473 K. In high-vacuum environments, hydrogen bonds dissociate progressively, enabling complete dehydration at 300 K. A series of intermediate metastable phases with unit cell volumes ranging from 1295–1412 Å^3^ (Cr-MIL-53) and 940–1212 Å^3^ (Ga-MIL-53) are captured, demonstrating continuous breathing behavior. The metal center dictates framework rigidity: Ga-MIL-53 delivers smaller unit cell volumes under identical hydration conditions due to stiffer metal–oxygen coordination bonds and a narrower deformation window, whereas Cr-MIL-53 possesses superior framework flexibility and undergoes more extensive pore expansion.^102^

Similarly, Co-TPDC-MOFs and Co-BDC-MOFs can serve as comparative models to uncover the atomic-scale breathing mechanism. Lithium storage in both materials relies on reversible Co^2+^/Co^0^ redox: Li^+^ first binds to CoO_6_ oxygen sites at 1.1 V and then to aromatic carbon sites at 0.7 V. XAS results prove that Co-TPDC-MOF fully reduces to Co^0^ during lithiation and recovers its original coordination upon delithiation, while Co-BDC-MOF only undergoes partial reduction and forms permanent atomic defects after cycling. Ligand sulfur dominates performance: thiophene groups in Co-TPDC-MOF create multi-dimensional Li^+^ transport channels, enabling weak, reversible Li–C–S–C interactions without Li trapping. By contrast, sulfur-free Co-BDC-MOF merely anchors Li *via* carboxyl groups and easily traps lithium ions. Kinetic data confirm that Co-TPDC-MOF exhibits a reduction peak *b*-value of 0.833 with capacitive-controlled fast Li storage and a post-cycling charge transfer resistance of only 123.64 Ω; Co-BDC-MOF delivers a lower *b*-value of 0.671 and a much higher resistance of 400.05 Ω, showing sluggish ion transport. DFT calculations of atomic lattice mechanics adopt the Hill approximation to calculate elastic moduli, and full-atom lattice relaxation simulations are carried out at gradient lithiation electron concentrations ranging from 0 to 2.0 e^−^/Co to extract lattice parameter variations of each crystal axis and plot volume change curves: Co-BDC-MOF undergoes dramatic anisotropic elongation along ligand-aligned *b* and *c* axes and irreversible lattice expansion with rising lithiation content, whereas Co-TPDC-MOF only generates slight uniform deformation without abrupt lattice collapse within 2.0 e^−^/Co and slight amorphization occurs gradually only under deep lithiation. Quantitatively, fully lithiated Co-TPDC-MOF expands by merely 4.04%, with a residual deformation of 0.42% after delithiation and a total volume variation of 1.04% over 700 cycles. Its capacity-normalized pressure reaches 3.34 MPa g(Ah)^−1^, far lower than that of lithium metal (15.86), silicon (8.97) and graphite (4.70), alongside a tiny electrode thickness fluctuation of 1.5 µm *versus* 6.5 µm for Li metal. At the atomic interface level, sulfur facilitates thin, stable SEI formation and suppresses accumulation of irreversible C–Li and lithium salt byproducts, yet Co-BDC-MOF suffers from continuous interfacial side reactions and rising impedance. These integrated atomic merits in coordination, ion transport, lattice mechanics and interfacial chemistry translate to outstanding macroscopic performance: the full cell retains 82% capacity after 700 cycles at 1.5 mA cm^−2^, and pouch cells operate stably for 50 cycles under a low stack pressure of 5 MPa. This comparative study verifies that the flexible breathing framework of sulfur-containing MOFs efficiently buffers lithiation-triggered volume expansion.^103^

Beyond flexible breathing frameworks that rely on reversible linker distortion to buffer volume strain, gate-type MOFs represented by the ZIF series achieve capacity–strain balance *via* sub-nanometer pore sieving and elastic metal–ligand bond deformation. For Si@ZIF-X, at the atomic scale, ZIF-8 contains two 1D atomic channels: 0.8 Å narrow 4-membered ring windows (matching Li^+^ ionic diameter) and 3.4 Å wide 6-membered ring channels. DFT data show that 4-MR delivers a strong adsorption energy of −3.43 eV for Li^+^(EC)_4_ solvated clusters, creating prominent desolvation to boost uniform Li^+^ transport without sacrificing Si's high theoretical capacity. Micron-sized rhombic dodecahedral ZIF-8 crystals act as elastic sacrificial buffers; the cycled XPS Zn 2p peak shift and slightly deformed ZIF polyhedrons in post-cycle SEM jointly confirm the strain-triggered atomic rearrangement of flexible Zn–N bonds to buffer silicon's large lithiation expansion. XPS confirms that no C–Si/N–Si covalent bonds form between the ZIF-8 shell and Si core, so buffering only relies on ZIF polyhedron elastic deformation. Electrochemically, Si@8Z retains 818.5 mAh g^−1^ after 650 cycles at 1 A g^−1^, while bare Si drops to 0 mAh g^−1^ merely after 200 cycles, fully resolving the capacity–volume contradiction.^76^ ZIF-67 also delivers a well-balanced trade-off between volume constraint and capacity retention. The ZIF-67 framework encapsulates silicon to physically confine and buffer silicon's nearly 300% volume expansion during lithiation. After cycling, the thickness of the Si@ZIF electrode is only 63 µm, much thinner than the 78 µm of the bare silicon electrode. The material features a hierarchical pore structure with a specific surface area of 1306.15 m^2^ g^−1^, which shortens lithium-ion diffusion paths and reduces polarization. Its micropores trap EC molecules to shrink the solvation shell of Li^+^, restrain continuous electrolyte decomposition and stabilize the SEI film. Paired with a self-supporting cellulose-carbon nanotube electrode with 73.4% porosity, the 3D conductive network exhibits unbroken electronic conduction, and internal pores accommodate volume variation for flexible applications. Benefiting from these synergistic effects, the half-cell delivers a stable capacity of 1744.6 mAh g^−1^ over 1000 cycles, while the full cell paired with LFP retains 80.5% of its initial capacity after 3400 cycles.^81^

*Ab initio* molecular dynamics simulations spanning tensile and compressive stresses from −2.75 to 50 GPa are adopted to systematically probe the intrinsic compressive mechanical behaviors of ZIF-8, which potentially uncovers the underlying mechanisms supporting the application feasibility of Si@ZIF-8 composites. Under hydrostatic compression, drastic distortion of ZnN_4_ tetrahedral units triggers a crystal-to-amorphous phase transition at 3 GPa, during which the Zn–N coordination number stays stable at 4. Sustained rupture of Zn–N bonds takes place above 10 GPa, and the Zn coordination number drops to 2.54 at 30 GPa alongside irreversible structural collapse of the framework. Pressure unloading before the critical amorphization threshold of 2.5 GPa induces an isostructural crystal–crystal phase transition *via* the swing effect of imidazolate linkers, corresponding to a decline of the linker *φ* angle from 19.8° to 10.6°. Pressure release from 3.5 GPa and 10 GPa separately produces low-density and high-density amorphous apZIF-8. For uniaxial compression, the critical pressure for amorphization increases to 4 GPa, and the generated amorphous phase features a density that is 20% higher than that formed under hydrostatic pressure. Complete framework breakdown emerges at a tensile load of −2.75 GPa. N–Zn–N bond angles are concentrated between 108° and 109° in crystalline ZIF-8, while they are broadly distributed from 90° to 150° in amorphous samples, with imidazole ring skeletons preserved under all pressure conditions. Quantitative mechanical characterization *via* simulations demonstrates that ZIF-8 has an average Young's modulus of 5.6 GPa and a compressive Poisson's ratio of 0.4, and its tensile strength is 50% higher than its compressive strength, presenting tensile-compressive anisotropy comparable to compact bone. The elastic constants *C*_11_, *C*_12_ and *C*_44_ are calculated to be 10.02 GPa, 7.69 GPa and 1.22 GPa, respectively, with a bulk modulus of 8.46 GPa, indicative of mechanical responses similar to those of flexible polymers.^104^ From the electronic perspective, crystalline ZIF-8 and amorphous apZIF-8 possess band gaps of 4.4 eV and 4.3 eV, respectively. Electronic properties are governed by imidazolate linkers, and crystal–amorphous phase transitions merely modify bond lengths and angles without compromising the material's inherent insulating performance. The reversible structural phase transition, low-modulus flexible skeleton, disparate tensile and compressive load-bearing capacities as well as stable insulating performance of ZIF-8 quantified by *ab initio* calculations collectively validate the mechanical buffering mechanism of Si@ZIF-8 composites. Silicon-based materials undergo dramatic volume expansion and accumulate massive internal compressive stress during lithiation and delithiation processes. Serving as the coating layer, ZIF-8 alleviates expansion-induced stress through reversible rotation of imidazolate linkers and deformation of ZnN_4_ tetrahedra, effectively suppressing pulverization and fragmentation of silicon particles. In addition, its robust insulating organic framework optimizes charge transport channels at the heterointerface, solidifying the structural compatibility and reliability of ZIF-8 as a stress-buffering matrix for silicon anodes.

Different from deformable flexible and gate-opening MOFs, rigid coordination networks sacrifice structural plasticity to gain superior framework stability under long cycling. The intrinsic rigid atomic mechanism is described as follows: metal cluster nodes and organic linkers form fixed spatial configurations *via* highly stable coordination bonds with barely tunable bond lengths and angles. Organic linkers cannot undergo large-scale rotation or slippage, and the whole framework is incapable of reversible global structural phase transitions. The geometric pore size and internal spatial configuration remain constant without atomic-level reversible framework reconstruction.

For M_3_(HHTP), during electrochemical cycling, the 2D planar coordination pore framework of Cu_3_(HHTP)_2_ exhibits outstanding structural stability. *In situ* XRD measurements reveal that the in-plane (*hk*0) diffraction peaks remain unchanged throughout lithiation and delithiation processes, while only the (001) peak corresponding to interlayer spacing shifts reversibly with a maximum interlayer expansion of merely 7%, demonstrating that the hexagonal microporous framework suffers no distortion or collapse. The interlayer regions, held together solely by weak π–π stacking interactions, possess intrinsic structural flexibility; Li^+^ insertion and extraction only induce minor reversible expansion/contraction of interlayer distance without irreversible breakdown of the planar framework.^105^ Conclusions from rigidity analysis are presented as follows: Ar sorption at 87 K confirms permanent porosity after activation; Co-CAT-1 and Ni-CAT-1 deliver BET surface areas of 490 m^2^ g^−1^ and 425 m^2^ g^−1^, respectively, with uniform hexagonal 12 Å pores retained without collapse. Synchrotron XRD and HRTEM verify invariant lattice parameters (*a* = 2.21 nm for Co-CAT-1, *a* = 2.19 nm for Ni-CAT-1) and constant interlayer distances of 0.32–0.33 nm upon desolvation, showing negligible structural shrinkage. The materials resist dissolution and morphological degradation in water and diverse organic solvents. They exhibit needle-like crystallite morphology after 85 °C hydrothermal synthesis and activation, and their honeycomb pore channels remain intact under TEM electron beam irradiation. Rietveld refinement of Ni-CAT-1 yields low *R*_wp_ = 8.54% and *R*_p_ = 6.33%, indicating highly ordered crystalline frameworks free from extensive structural defects or collapse.^106^

For M_4_(*o*-TTFOB)(bpm)_2_(H_2_O)_2_, single-crystal structural analysis reveals that bpm forms rigid five-membered chelate rings with metal centers to lock the M–N coordination geometry. The octacarboxylate *o*-TTFOB ligand simultaneously bridges up to ten metal ions to construct a multidirectionally crosslinked M–O coordination network. Combined with the exceptional framework rigidity validated by acid–base tolerance, solvent resistance and thermogravimetric measurements, the multiple coordination restraints impose substantial structural distortion barriers. At the atomic scale, effective free rotation of M–N and M–O coordination bonds is therefore prohibited at ambient temperature. Such robust rigid skeletons can preserve abundant uncoordinated carboxylate oxygen sites for lithium binding and sustain unobstructed ion migration pathways during repeated lithiation/delithiation, synergistically boosting reversible Li^+^ storage capacity and fast diffusion kinetics.^107^

Similarly, for Cu-DBC, each Cu^2+^ center forms fused five-membered O–Cu–C–C–O chelate rings with catechol groups of contorted DBC ligands, fixing Cu–O bond lengths and bite angles at the atomic scale to block free torsion around Cu–O axes. Combined with four-fold interpenetrated multidirectional crosslinking of rigid fully fused aromatic linkers, the whole coordination network generates prominent steric and electrostatic restraints against lattice distortion during ion insertion/extraction. *Ex situ* PXRD verifies complete retention of crystal lattice after thousands of sodiation–desodiation cycles. Benefiting from this atomically rigid configuration, Cu-DBC achieves a reversible capacity of 120.6 mAh g^−1^ at 0.05 A g^−1^, with only 18.1% capacity attenuation after 4000 cycles at 2 A g^−1^, realizing highly stable alkali-metal ion storage.^108^

In summary, conductive MOFs constructed *via* the extended conjugation pathway feature atomically locked rigid planar frameworks with non-flexible geometries resistant to deformation. Their stable spatial arrangement sustains extensive orbital overlap between metal d-orbitals and ligand π-orbitals while fixing interlayer π–π stacking distances, establishing continuous charge transport pathways that serve as the core structural prerequisite for high electrical conductivity. Relevant experimental data from the literature indicate that single crystals of rigid two-dimensional Ni_3_(HITP, 2,3,6,7,10,11-hexaiminotriphenylene)_2_ exhibit an in-plane conductivity up to 150 S cm^−1^, and thin-film Cu_3_(HOTP, 2,3,6,7,10,11-hexahydroxytriphenylene) conductive MOFs reach a conductivity of 0.29 S cm^−1^. Distortions of the framework geometry or shifts in interlayer spacing weaken orbital coupling, leading to conductivity degradation by orders of magnitude. Consequently, such rigid conductive MOFs face a trade-off between pore volume and electronic conduction. Further in-depth research is required to simultaneously achieve high pore volume and outstanding electronic conductivity.^109^

**Fig. 10:**
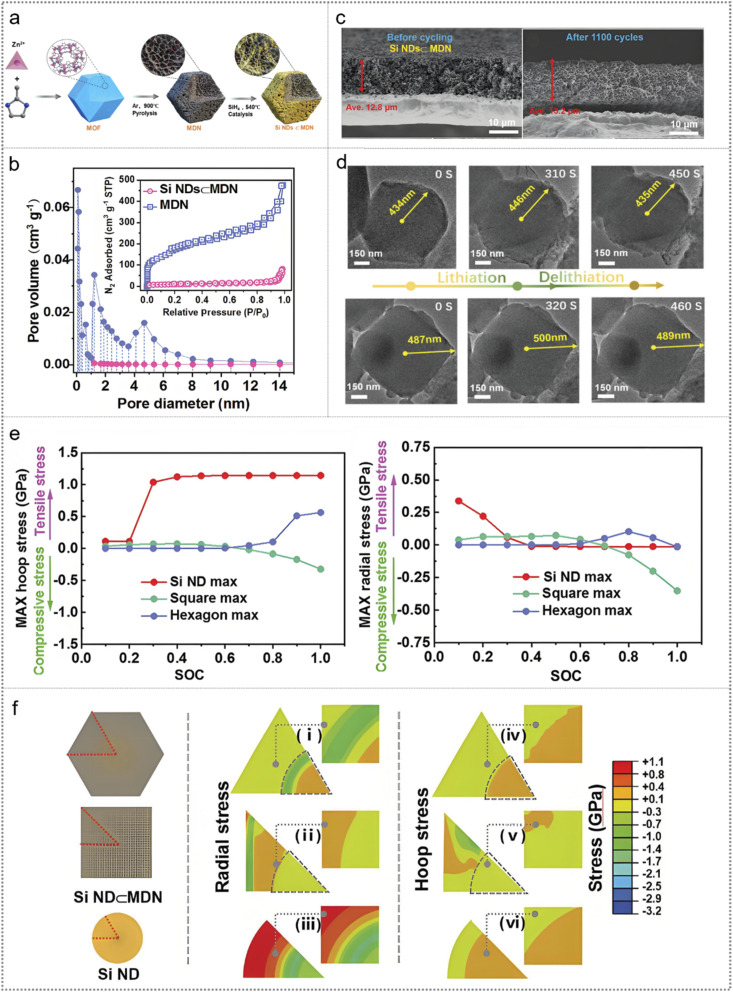
Si NDs ⊂ MDN silicon anodes: fabrication, structure and stability.^99^ Adapted/reproduced from ref. 99 with permission from Wiley, copyright 2022. (a) Synthesis schematic: MOF pyrolysis (900 °C, Ar) yields MDN, with Si NDs grown in pores *via* SiH4. (b) N_2_ sorption confirms that MDN has a hierarchical micro–mesoporous structure, while Si NDs ⊂ MDN shows near-zero uptake/porosity, verifying confined Si encapsulation. (c) Cross-sectional SEM shows that electrode thickness only increases from 12.8 µm to 13.2 µm after 1100 cycles, confirming ultra-low volume expansion. (d) *In situ* TEM reveals a ∼2.76% volume change during lithiation/delithiation, confirming zero-strain behavior. (e) Stress analysis shows that Si NDs ⊂ MDN has far lower hoop/radial stress than pure Si NDs, resolving the volume expansion–capacity trade-off. (f) Finite element simulation models of Si NDs ⊂ MDN and bare Si ND showing stress evolution during the lithiation process. Stress distributions of the sector area of the three models during the lithiation process. The steps (i–vi) correspond to radial and hoop stress values for the three models, respectively. Bottom panels depict the sector area of the three models; top panels are enlarged views of the regions marked by rectangles in (i–vi).

Though pristine MOFs (flexible, gated, and rigid types) tune electrode volume through intrinsic coordination framework design, their high intrinsic resistance restricts practical application. Pyrolyzed MOF-derived carbon materials break this limitation *via* a carbon confinement strategy. Taking the above-mentioned ZIF-8 derivatives as typical cases: for Si@MOF derivatives, one of the examples mentioned above, ZIF-8 was pyrolyzed at high temperature to fabricate a porous carbon nanoreactor (MDN); under negative pressure, SiH_4_ catalytically grew silicon nanodots (Si NDs) in MDN pores to yield Si NDs ⊂ MDN, with the fabrication and structure shown in Fig. 10a. The hierarchical porous structure of metal-doped MDN exerts excellent confinement: the carbon matrix's mechanical stability offsets Si's lithiation expansion, while size-matched pores inhibit Si ND agglomeration and ensure Li^+^ diffusion. N_2_ adsorption–desorption isotherms and pore size distribution (Fig. 10b) confirm that pure MDN has a well-developed micro–mesoporous hierarchical structure and high porosity, while Si NDs ⊂ MDN exhibits nearly zero N_2_ uptake and pore volume, directly verifying full pore filling by Si NDs, confined Si encapsulation, and effective suppression of volume expansion for a stable electrode structure. This anode shows exceptional structural stability with a <3% zero-strain characteristic: the thickness only increased from 12.8 µm to 13.2 µm after 1000 cycles (Fig. 10c). *In situ* TEM (Fig. 10d, under a ± 3 V bias, showing hexagonal/square views during lithiation–delithiation) confirms a volume change rate of ∼2.76%. Stress analysis (Fig. 10e) reveals that Si NDs ⊂ MDN has far lower maximum radial/circumferential tensile stress (0.1/0.5 GPa) than pure Si NDs (0.4/1.2 GPa). Furthermore, bare Si nanodots (Si ND) and Si NDs ⊂ MDN composites were set as controls. Three finite element models, bare Si ND, Si NDs confined in hexagonal pores, and Si NDs confined in square pores, were built to simulate stress evolution during lithiation (Fig. 10f). Bare silicon generates intense internal tensile stress (marked by extensive red regions) upon lithiation, which readily induces crack propagation and particle pulverization. By contrast, the outer shell derived from MOFs can drastically mitigate and even eliminate destructive tensile stress inside silicon particles, stabilizing the overall granular structure.^99^ Similarly, after pyrolysis at 700 °C under an N_2_ atmosphere, the ZIF-8-derived carbon matrix achieves atomic-scale tight anchoring of silicon nanoparticles *via* interfacial C–Si and N–Si bonds, as verified by XPS peak deconvolution, restricting the long-range migration of Si atoms during alloying and alleviating the drastic ∼300% volumetric expansion of silicon. The mesoporous voids with a size range of 2–10 nm generated by framework collapse accommodate the volume expansion of silicon; the specific surface area decreases from 1070 m^2^ g^−1^ of pristine Si@ZIF-8 to 351 m^2^ g^−1^ after pyrolysis, while interlayer sliding of carbon atomic stacks enables reversible atomic-level flexible deformation to dissipate interfacial mechanical stress, averting bond fracture at Si–carbon heterointerfaces and electrical isolation of active grains. In addition, homogeneously dispersed Zn sites (30.7 wt%) and N heteroatomic defects construct uninterrupted electronic conduction pathways. Benefiting from such atomic-scale structural regulation, the composite delivers a high reversible capacity of 1050 mAh g^−1^ at 50 mA g^−1^ and retains 830 mAh g^−1^ over 500 cycles at 200 mA g^−1^, enabling full utilization of lithium storage active sites while buffering volume fluctuation and resolving the inherent trade-off between high theoretical capacity and severe volume expansion of silicon anodes.

Collectively, flexible skeletons, gated pore structures, rigid coordination backbones and carbonized MOF derivatives represent four typical structural routes to balance capacity and volume fluctuation for Li^+^ storage. Future research should further focus on resolving the inherent trade-off between electronic conductivity and structural deformability to develop high-performance electrode materials with both fast charge transport and outstanding anti-distortion capability.^110^

**Fig. 11:**
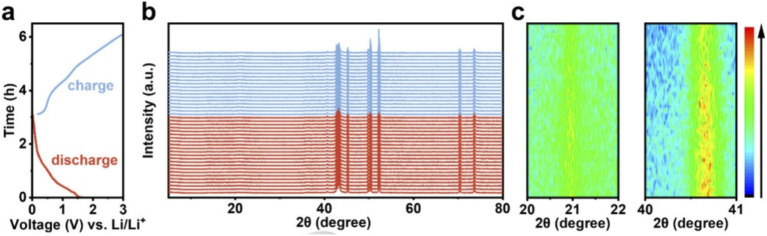
(a–c) *In situ* XRD curves and corresponding charge/discharge curves of Si–Ni–HHTP.^92^ Adapted/reproduced from ref. 92 with permission from American Chemical Society, copyright 2025.

*In situ* characterization enables real-time tracking of shifts and intensity fluctuations in characteristic signals to resolve the intrinsic breathing behavior of MOFs. However, dedicated experimental explorations targeting this phenomenon remain largely underexplored. Representative advanced cases are presented below:

The application of Si–M-HHTP (M = Co, Ni, Cu) in LIBs was examined. To clarify how conductive M-HHTP regulates the Li^+^ storage mechanism of nanoSi, *in situ* XRD measurements were carried out on the Si–Ni-HHTP composite. Fig. 11a displays its galvanostatic charge/discharge profiles measured between 0.005 V and 3 V, and Fig. 11b collects the corresponding XRD diffraction patterns recorded at different cut-off potentials. The diffraction signals at 45.2°, 50.3°, and 52.2° belong to Be foil; peaks at 42.7°, 70.3°, and 73.5° correspond to BeO and Li foil, respectively; and the peak at 43.1° arises from Cu foil. These substrate-related components are electrochemically inert and do not engage in redox reactions during cycling. No diffraction peaks of elemental silicon can be detected, mainly because silicon transforms into amorphous Li_*x*_Si_*y*_ alloys after the initial two lithiation cycles, and this amorphous phase persists in subsequent charge and discharge processes. Contour maps collected over the 20–22° and 40–41° angular ranges are presented in Fig. 11c. Obvious peak shifts near 21° and 40.7° are observed for Ni–HHTP, directly reflecting its reversible lithiation/delithiation redox behavior. This evidence confirms that the M–HHTP framework possesses a certain degree of structural reversibility.

**Fig. 12:**
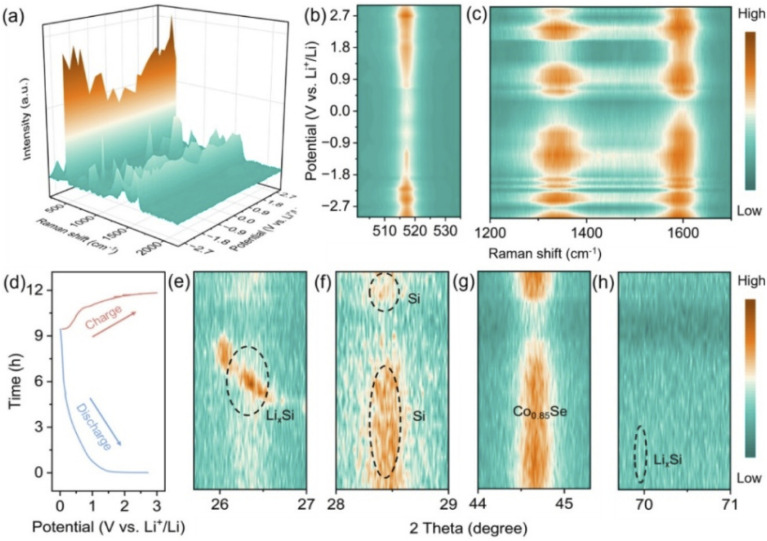
(a) *In situ* 3D Raman map and (b and c) contour plots of the Si@Co_0.85_Se/NC anode during lithiation/delithiation. (d–h) *In situ* XRD patterns of Si@Co_0.85_Se/NC during charging and discharging.^111^ Adapted/reproduced from ref. 111 with permission from American Chemical Society, copyright 2026.

In addition to pristine Si@MOF composites, *in situ* characterization was also conducted to elucidate the electrochemical evolution of Si@MOF-derived carbon anodes. Porous silicon is coated with MOF-derived Co_0.85_Se, and the as-obtained Si@Co_0.85_Se composite is fully encapsulated inside an NC matrix. *In situ* characterization was implemented to monitor its structural evolution during electrochemical cycling. The *in situ* 3D Raman spectrum in Fig. 12a records the real-time variation of characteristic peaks at different potentials, and the corresponding contour plots display the Si characteristic peak at 517.3 cm^−1^, alongside carbon D and G bands at 1335.4 cm^−1^ and 1591.4 cm^−1^ (Fig. 12b and c). During discharge, lithiation weakens the signal of silicon, and Li^+^ intercalation simultaneously lowers the intensity of D and G bands. In the following delithiation step, both Si and carbon characteristic peaks gradually recover their original intensity, proving favorable structural dynamic stability of the composite throughout charge and discharge. *In situ* XRD patterns further validate its reversible structural evolution (Fig. 12d–h). As lithiation proceeds, the diffraction peaks of Si (28.4°) and Co_0.85_Se (44.7°) gradually fade away, while new peaks assigned to Li_*x*_Si alloys arise at 26.4° and 69.8°. At the discharge cutoff of 0.01 V, the diffraction signals of Si and Co_0.85_Se become faint, whereas the peaks of Li_*x*_Si alloys are markedly intensified. Upon charging, the characteristic peaks of Si and Co_0.85_Se reappear, manifesting reversible delithiation behavior. The inconsistent positions of Li_*x*_Si diffraction peaks shown in Fig. 12e and h mainly stem from the kinetic discrepancy between Li^+^ insertion and extraction.

#### 4.2.2 Regulation of SEI film stability enabled by MOF-based scaffolds

With atomically regular pores and abundant coordination sites, MOFs enable precise sieving of Li^+^ ions while blocking solvent molecules, thereby fundamentally avoiding the formation of inferior SEI films induced by disordered electrolyte reduction. Meanwhile, the metal nodes and organic functional groups of MOFs can guide uniform Li^+^ deposition at the atomic scale to restrain lithium dendrite growth and construct thin, continuous and flat SEI interphases. Benefiting from the unique interfacial confinement effect of MOFs, the kinetics of SEI-forming reactions can be modulated to generate compact passivation layers rich in stable inorganic species and significantly mitigate persistent interfacial side reactions. In addition, atomically ordered pore channels confine ion transport pathways to prevent excessive local thickening and cracking of SEI films, stabilizing electrode interphases during long-term battery cycling. Typical examples are presented as follows:

Breathable MOFs represented by the MIL-53 series possess thermodynamically reversible flexible one-dimensional channel frameworks with obvious breathing behavior, which can synchronously expand and contract following the volume fluctuation of silicon during lithiation and delithiation. This real-time adaptive elastic buffering avoids repeated cracking, peeling and regrowth of the SEI film caused by silicon particle pulverization, and maintains complete electrode–electrolyte interface all the time. Meanwhile, their dynamically tunable pore channels homogenize lithium ion flux and optimize solvent desolvation, inhibiting violent and uncontrolled electrolyte reduction on the bare silicon surface, thus restraining abnormal continuous thickening of the SEI and effectively stabilizing the interfacial SEI film over long cycles.^69^ Apart from flexible breathing MOFs, cage-type zeolitic MOFs represented by the ZIF series achieve SEI regulation *via* molecular sieving effects.

For Si@ZIF-8, ZIF-8 exerts a size-sieving effect *via* its sub-nanometer windows (∼4.2 Å) and microporous structure, accelerating Li^+^ desolvation, repelling solvent molecules and mitigating side reactions that cause abnormal SEI growth. Its internal cavities act as anion reservoirs to enrich TFSI^−^ and other anions, modulate electrolyte decomposition pathways, and induce the formation of a dense, uniform, LiF/Li_3_N (or Li_2_CO_3_)-rich stable SEI. Additionally, ZIF-8's ordered porous structure and high mechanical strength ensure uniform Li^+^ flux distribution, preventing local SEI cracking from uneven ion concentrations; lone pair electrons on the imidazole ring nitrogen atoms facilitate charge transfer and the rapid formation of stable SEI components. It also boosts interfacial compatibility with electrolytes/electrodes, enabling uniform SEI coating and lowering detachment risk, ultimately realizing the synergistic effect of the SEI membrane's “component optimization, structural integrity, and efficient transport”.^72,112^ Unlike the flexible ZIF and MIL-53 series, rigid non-breathing MOFs rely on static physical confinement to stabilize electrode interphases, as exemplified by MIL-100 (Fe).

MIL-100(Fe) is a rigid, non-breathing iron-based porous MOF. Constructed from trinuclear iron-oxygen clusters and 4,5-imidazole dicarboxylate ligands, its stable cage framework resists deformation caused by lithium ions and electrolytes. After being *in situ* coated on Si/Ag@PM and etched with dilute hydrochloric acid to remove iron oxide by-products, it forms a porous outer shell with a pore size of about 4 nm. The dual-layer structure of an inner PM conductive buffer and an outer rigid MIL-100 shell realizes effective SEI stabilization. The MOF shell acts as a physical barrier to limit electrolyte contact with silicon, so the SEI only forms in the first cycle with no ongoing side reactions. Its uniform mesopores induce the formation of a compact thin SEI film, increasing the initial coulombic efficiency to 80.4%. The rigid framework, interlayer voids and inner carbon network accommodate silicon's volume expansion during cycling and prevent repeated damage and regeneration of the SEI. Meanwhile, nanosilver, carbon network and MOF channels optimize electron and ion transport, reduce electrode polarization and inhibit extra electrolyte decomposition, ensuring long-term SEI stability. After 500 cycles at 1 A g^−1^, the composite delivers a discharge capacity of 615 mAh g^−1^ and 62.9% capacity retention, with coulombic efficiency approaching 100% during long cycling. It verifies that MIL-100(Fe) can sustainably regulate and stabilize the SEI film.^113^

**Fig. 13:**
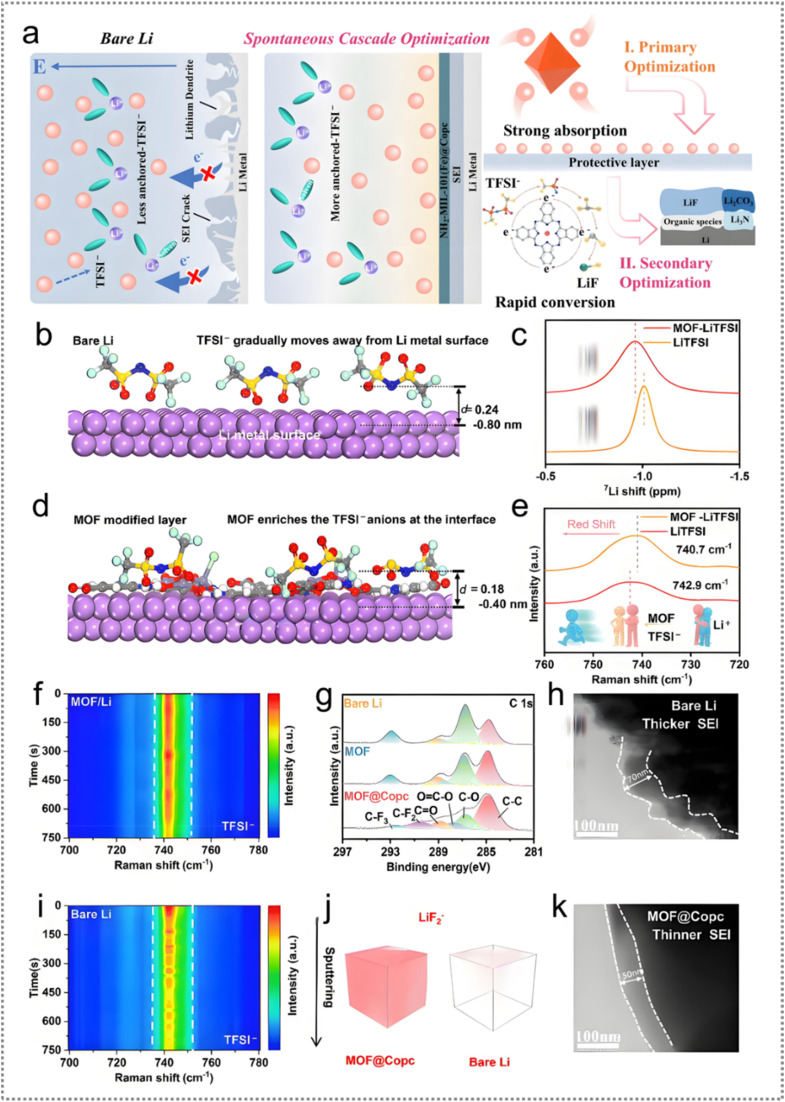
Mechanism and multi-scale characterization of the spontaneous cascade optimization strategy based on NH_2_-MIL-101(Fe)@Copc for regulating the SEI of lithium metal anodes.^114^ Adapted/reproduced from ref. 114 with permission from Royal Society of Chemistry, copyright 2025. (a) Schematic of the spontaneous cascade optimization mechanism, comparing the interfacial behaviors of the bare Li anode (lithium dendrite growth, SEI cracking, and free TFSI^−^) and MOF-modified Li anode (TFSI^−^ anchoring and enrichment and formation of a LiF/Li_3_N-rich SEI). (b and d) Adsorption models of TFSI^−^ on bare Li and MOF-modified Li surfaces, confirming that MOF anchors TFSI^−^ in the vicinity of the Li surface (0.18–0.40 nm), which is far superior to bare Li where TFSI^−^ is repelled and distant (0.24–0.80 nm). (c) ^7^Li NMR spectra and (e) Raman spectra, verifying the strong MOF–LiTFSI interaction *via* a characteristic peak redshift. (f and i) Time-resolved *in situ* Raman spectra, confirming that the MOF-modified layer restricts TFSI^−^ migration and achieves continuous interfacial anion enrichment. (g) C 1s XPS spectra, verifying more complete LiTFSI decomposition and significantly increased LiF/Li_3_N contents in the SEI after MOF@Copc modification. (h and k) Cryo-electron microscopy characterization, comparing SEI microstructures of bare Li (70 nm thick, heterogeneous SEI) and MOF-modified Li (50 nm thick, uniform and dense SEI). (j) Schematic of TOF-SIMS depth analysis, confirming uniform LiF distribution along the depth in the modified SEI.

Similar studies have proposed a spontaneous cascade optimization strategy for NH_2_-MIL-101(Fe)@Copc, which anchors and enriches interfacial TFSI^−^ through Fe^3+^-mediated Lewis acid–base interactions and amino hydrogen bonds to optimize the SEI. Fig. 13a schematically shows that Fe^3+^ with empty orbitals interacts with TFSI^−^*via* Lewis acid–base and hydrogen-bonding effects, anchoring free TFSI^−^ on the modified layer for preliminary interfacial anion enrichment. Molecular dynamics (Fig. 13b and d) confirm MOFs anchor TFSI^−^ near the Li surface and increase its coordination number. NMR and Raman (Fig. 13c,e) spectra verify the strong MOF–LiTFSI interaction *via* spectral redshifts. *In situ* Raman spectroscopy (Fig. 13f and i) proves that the MOF layer restricts TFSI^−^ migration and achieves interfacial enrichment. Further multi-scale characterization validates SEI optimization: XPS C 1s spectra confirm that MOF@Copc modification significantly enhances C–F_3_/C–F_2_ species and optimizes oxygen-containing functional groups, accelerating fluorinated additive (FEC/LiBOB) decomposition *via* Lewis acid–base interactions or catalysis to form a stable LiF-rich SEI (Fig. 13g); TOF-SIMS (Fig. 13j) reveals uniform LiF distribution in the SEI; cryo-electron microscopy (Fig. 13h and k) shows that the modified 50 nm uniform and dense SEI is superior to bare Li's 70-nm inhomogeneous SEI, effectively regulating Li deposition and ion transport to stabilize the Li-metal anode interface.^114^

For MOF derivatives, professor Gao's team-developed a Si@C-ZIF electrode that precisely modulates the SEI film of lithium-ion batteries *via* the structure–performance synergy of ZIF-67-derived carbon shells. At the atomic scale, bare silicon surfaces possess abundant unsaturated Si dangling bonds with strong reducing activity, which spontaneously trap EC/DEC solvent molecules in the electrolyte and trigger uncontrolled continuous irreversible reduction, leading to repeated generation of loose, thick SEI films and massive loss of active lithium. The *in situ* formed ZIF-67-derived nitrogen-doped carbon shell tightly encapsulates silicon nanoparticles and passivates these reactive Si dangling bonds at the atomic level to cut off spontaneous solvent decomposition on silicon substrates. Meanwhile, electronegative N heteroatomic sites distributed uniformly within the carbon framework create local electron-rich microdomains that rearrange the solvation sheath of Li^+^, lower the reduction energy barrier of carbonate solvents, and guide the electrolyte to undergo mild, uniform reduction rather than violent random decomposition on bare silicon, consequently generating a thin, rigid SEI film dominated by inorganic LiF and Li_2_O with ordered atomic stacking and unobstructed ion transport channels. In addition, the sp^2^-hybridized carbon skeleton of the outer shell can absorb the lattice strain caused by atomic-scale stretching and breaking of Si–Si bonds during lithiation and delithiation through C–C bond torsion and interlayer slippage, preventing atomic-level cracking and reconstruction of the preformed SEI film; the embedded Co nanoparticle atomic catalytic sites further promote the conversion of electrolyte reduction products into LiF-rich inorganic phases, whose rigid atomic layer structure effectively blocks solvent molecules from penetrating the SEI to corrode internal silicon atoms. Furthermore, the 1–4 nm micro–mesopores on the carbon shell screen solvated Li^+^ at the atomic scale, shorten the Li^+^ diffusion path, limit long-term retention of excess electrolyte at the electrode interface, and fundamentally eliminate continuous abnormal thickening of the SEI, which raises the initial coulombic efficiency from 36% of bare silicon to 70% and greatly stabilizes the electrode–electrolyte interface over long cycles.^40^

All aforementioned MOF-based materials modulate SEI interphases through three core synergistic pathways, despite their distinct structural characteristics. In general, breathable MOFs dominate dynamic adaptive SEI stabilization *via* reversible framework breathing; gate-opening MOFs optimize SEI composition and uniformity *via* size sieving and functional modulation; rigid MOFs stabilize interfaces through static physical confinement and chemical adsorption; and MOF-derived carbon derivatives realize root-level passivation and long-term SEI stability through defect elimination, catalytic regulation, and structural buffering.^114–117^

### 4.3 Rational design and performance optimization of Si@MOF-based composite anodes

MOF-silicon composites provide effective solutions to the challenges facing Si-based anodes. Rational material selection and composite design are essential to fully exploit the advantages of both components, and multiple strategies can be synergistically applied to achieve optimal performance.

**Fig. 14:**
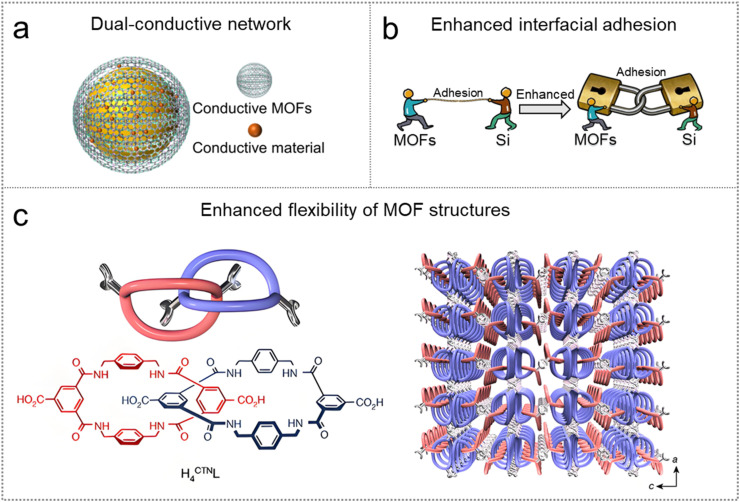
Schematic diagrams of multi-dimensional structural enhancement mechanisms for silicon-based anodes. (a) Dual-conductive network: a composite 3D network constructed from conductive MOFs and conductive materials. (b) Enhanced interfacial adhesion: MOF and Si are bonded by strong chemical bonds instead of weak physical contact, significantly improving interfacial adhesion. (c) Enhanced flexibility of MOF structures: the schematic diagram of the flexible MOF crystal structure based on ligand H_4_CTNL, showing high structural flexibility.^118^ Adapted/reproduced from ref. 118 with permission from Springer Nature, copyright 2021.

The optimization of electronic and ionic conduction properties is the core prerequisite for high-performance Si@MOF anode materials (Fig. 14a). Dual conductivity optimization, interfacial bonding reinforcement, elastic skeleton engineering, crystal engineering, *etc.*, can be adopted to comprehensively promote the electrochemical performance of Si@MOF composites.

Concerning the construction of conductive pathways. For example, precise S/Se/Te doping into HHS ligands, combined with ligand synthesis, solvothermal assembly, and slipped AA’ stacking regulation, allows the fabrication of 2D Cu-X-HHS MOFs. Increasing the chalcogen atomic number optimizes ligand–metal orbital matching to enhance bonding-mediated charge transfer; reduces interlayer spacing, with Te in Cu-Te-HHS forming 3.67 Å contacts to boost interlayer spatial charge transport *via* lone-pair electrons and π–π stacking; and narrows the band gap to 0.72–0.93 eV and strengthens conduction band dispersion, with Cu-Te-HHS showing metallic bands across the Fermi level. Accordingly, Cu-Te-HHS achieves a room-temperature conductivity of 3.21 S cm^−1^ (10^4^ × 100 × that of Cu–S/Se-HHS), and its carrier mobility and concentration are 29× and two orders of magnitude higher than those of Cu-S-HHS.^119^ Other studies have proposed similar methods to enhance the conductivity of MOFs, such as introducing iodine/polyiodides, conductive polymers, *etc*., *via* porosity of MOFs. These guest molecules disperse uniformly in the pores, filling gaps in the MOF framework's conductive pathways to boost conductivity.^120^

Fig. 14b depicts the mechanism of interfacial bonding reinforcement. Rather than relying on conventional physical adhesion, MOFs can be engineered to form robust chemical lock-and-key interactions with silicon *via* surface modification and *in situ* growth strategies, which greatly enhance interfacial affinity and structural integrity. A representative example is surface functionalization: the terminal –NH_2_ groups of 3-aminopropyltriethoxysilane undergo amidation with the –COOH moieties of CuSn-BTC ligands to generate O–C–N covalent linkages. Subsequent strong coordination between the organic linkers and Cu^2+^/Sn^2+^ ions achieves intimate integration between MOFs and SiO_*x*_ at the molecular scale, alongside homogeneous dispersion of Cu and Sn species. Such robust interfacial interactions stabilize the SiO_*x*_ framework, accelerate charge transport, activate electrochemically inactive phases for elevated specific capacity, improve cycling durability, and hinder the agglomeration of metal active sites.^121^ Similarly, Ketjen black-derived oxidized carbon materials are adopted to realize interfacial reinforcement and boost the reaction kinetics of electroreductive C–C coupling of benzaldehyde, shortening the interfacial distance between copper phthalocyanine and the carbon support from 3.70 Å to 3.26 Å. The energy barrier of the rate-determining coupling step decreases from 0.79 eV to 0.53 eV, affording a hydrobenzoin production rate of 0.62 mmol h^−1^ cm^−2^ and a high faradaic efficiency of 83.4%. Moreover, such a modified catalytic system delivers universal performance improvement for various multi-electron reactions including nitrobenzene coupling and CO_2_ electroreduction.^122^

Ligand engineering can enable the enhancement of breathing behavior in MOFs.^123^ For instance, replacing MOF-5's linear 1,4-bdc with curved 1,3-bdc to build a topologically aperiodic fcu-6 network (*e.g.*, TRUMOF-1) blocks stress transmission, activates multi-local deformation for stress absorption, and enhances static disorder to form a pressure buffer, improving MOF elastic stability.^124^ In addition, the [2]catenane ligand (H_4_^10N^L) coordinates with Co^2+^ to construct flexible frameworks *via* mechanical interlocking of catenane units rather than rigid covalent linkages (Fig. 14c). Treatment with DMF/THF further attenuates hydrogen bonding between adjacent macrocycles, reducing overall framework rigidity. From a mechanistic perspective, such interlocked structures allow the interwoven macrocycles to slide along the crystallographic *c*-axis upon external compression, achieving a reversible volume contraction of 5% under 0.88 GPa. The disruption of inter-macrocycle hydrogen bonds by solvent molecules yields a low Young's modulus ranging from 1.63 to 1.77 GPa. This anisotropic deformation predominantly occurring along the *c*-axis maintains intact framework architecture and facilitates efficient stress dissipation, which collectively endows the material with superior structural flexibility.^118^

Besides, crystallization modulation balances the capacity–expansion trade-off of Si@MOF anodes. Si@cobalt-ZIF-62 glass is fabricated *via* reflux at 130 °C for 48 h and subsequent Ar-protected melt-quenching. Silicon nanoparticles function as heterogeneous nucleation sites for *in situ* crystalline ZIF-62 encapsulation. Heating to 450 °C at 10 °C min^−1^ with a 5 min hold melts the outer ZIF layer, and Ar-assisted natural cooling converts molten ZIF into amorphous glass while retaining crystalline silicon cores. The glass framework features distortable Co–N bonds, slidable organic rings and inherent nanoscale voids, which atomically buffer silicon's over 300% volume variation and inhibit particle pulverization. Disordered glass supplies abundant Li^+^ diffusion channels and redox sites, and synergistic lithium storage of silicon and ZIF glass elevates electrochemical properties. This synthesis relies on conventional laboratory devices and can be generalized to Sn- and SnO_2_-based anodes.^125^

To develop Si@MOF anodes with high capacity, low volume expansion and enhanced stability, enhancing conductivity, strengthening interfacial interaction, and providing elastic confinement buffering are core optimization strategies. Beyond the explored technical routes, their related method systems still have great untapped space, offering substantial potential for high-performance material development.

Apart from Si@MOF materials, the design strategies for Si@MOF derivatives are listed as follows:

The *in situ* coating strategy involves dispersing Si nanoparticles in MOF precursor solutions, allowing MOFs to nucleate and grow directly on the Si surface through coordination between metal ions and organic ligands. This yields a uniform, tightly bonded Si@MOF core–shell structure with precisely tunable shell thickness and compactness. For example, a ZnO seed layer was deposited on Si to guide ZIF-8 growth, and subsequent pyrolysis produced an N-doped carbon/CNT network with high conductivity and flexibility.^126^ In another design, Co^2+^ adsorption on Si in methanol promoted ZIF-67 nucleation, and pyrolysis at 600 °C formed hollow Si@Co–NC with enhanced mass transport enabled by N/Co co-doping.^42^

**Fig. 15:**
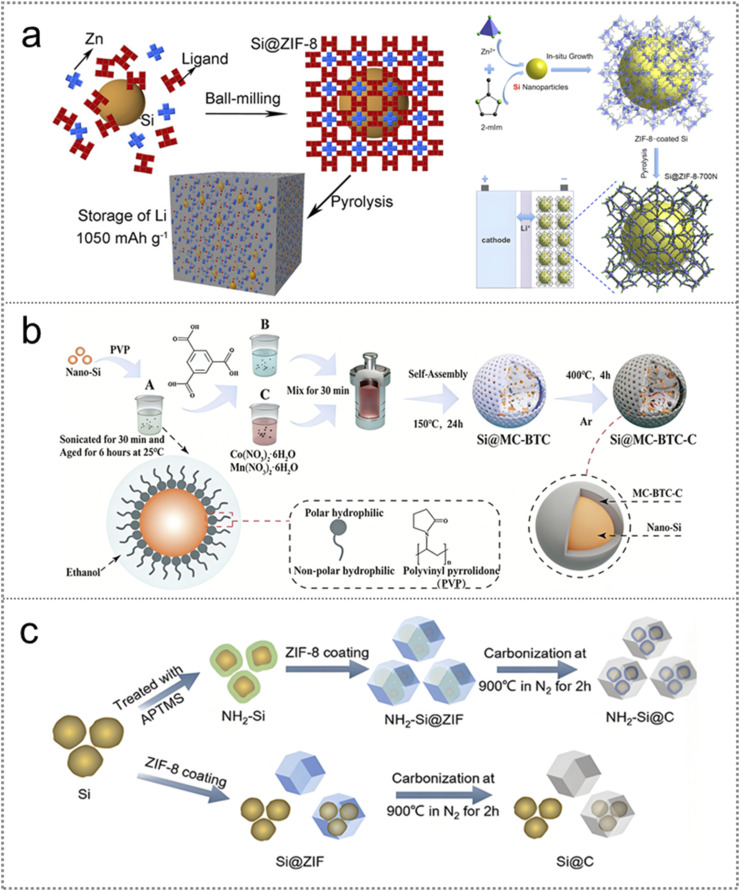
Schematic fabrication processes of three silicon-based anodes for lithium-ion batteries. (a) Ball-milling and *in situ* growth of Si@ZIF-8, followed by pyrolysis.^73^ Adapted/reproduced from ref. 73 with permission from American Chemical Society, copyright 2015. (b) Self-assembly and pyrolysis for the Si@MC-BTC-C core–shell structure.^89^ Adapted/reproduced from ref. 89 with permission from Wiley-VCH Verlag, copyright 2025. (c) Fabrication of amino-modified (NH_2_–Si) and unmodified Si@ZIF-8-derived carbon-coated Si anodes.^96^ Adapted/reproduced from ref. 96 with permission from Elsevier, copyright 2025.

Fig. 15a illustrates the *in situ* growth mechanism of Si@ZIF-8: continuous uniform ZIF-8 shells are grown on Si *via in situ* coordination self-assembly, forming ZIF-8-encapsulated Si; pyrolysis converts ZIF-8 into N-doped porous carbon, yielding Si@ZIF-8-700N.^73^ Yuan *et al.* prepared Si@MC-BTC *via in situ* solvothermal growth of a Mn/Co bimetallic MOF, and controlled pyrolysis generated a carbon/MnO/CoO buffer layer for synergistic stabilization (Fig. 15b).^89^ In this strategy, MOF shells uniformly encapsulate Si and, upon pyrolysis, form porous carbon networks doped with metal/metal oxides and nitrogen species. These structures exhibit excellent electrical conductivity and regulate interfacial contact and mass transport, and can be further combined with other methods for performance enhancement.^127^ In addition, Zn-, Al-, or Mg-based MOFs can be converted into metal-free porous carbons with a high surface area and tunable hierarchical porosity by thermal evaporation or acid etching.^128^ Heteroatom doping further improves conductivity and provides active sites (*e.g.*, at 700 °C under a reducing atmosphere, Se vapor reacts with Zn from decomposed ZIF-8 to form ZnSe, while precursors carbonize into carbon fibers wrapping ZnSe).^129^ Meanwhile, bimetallic MOFs such as ZnCo-ZIF and Ni-MOF yield carbon matrices anchored with single-atom sites (*e.g.*, Fe–N_4_ and Co–N_4_) or metal nanoparticles, which synergistically accelerate electron transport and reinforce structural stability.^127^

In addition to mainstream composite routes, surface functionalization serves as an effective auxiliary approach. By modifying Si surfaces with amino groups (*e.g.*, APTMS) or polymers (*e.g.*, PVP), interfacial coordination between Si and MOFs is strengthened, improving dispersion, uniformity, and structural integrity after carbonization. For instance, PVP was adsorbed on Si nanoparticles to improve dispersion and bridge the Si–MOF interface, enabling directional growth of ZIF-67 into a hierarchical porous structure.^81^ In another example, Si was aminated with APTMS to form NH_2_–Si, which promoted uniform assembly of ZIF-8 on the Si surface. Pyrolysis at 900 °C then produced a N-doped carbon-encapsulated Si composite (NH_2_–Si@C, Fig. 15c).^96^

To sum up, common fabrication strategies for Si@MOF derivatives mainly include *in situ* MOF coating followed by pyrolysis, metal/nonmetal integration modification, and silicon surface pre-functionalization to optimize interfacial combination, so as to achieve balanced volume variation and high capacity as well as improved conductivity.

## 5. Critical gaps in the practical deployment of Si@MOF materials

MOFs provide an innovative solution to the inherent capacity–volume expansion contradiction in silicon-based anodes. Nevertheless, the commercialization of Si@MOF composites remains hindered by multiple bottlenecks.

Current research on silicon-based MOF (Si@MOF) composites still relies heavily on trial-and-error strategies and lacks quantitative theoretical guidance. To date, several fundamental bottlenecks remain to be urgently resolved: pore size matching between Si particles and MOFs, the modulation mechanism of metal nodes governing SEI film formation, and optimization of interfacial electronic conductivity between Si and MOFs. These issues prevent the reliable reproduction of high-capacity and long-cycle performance achieved in laboratories, and hinder the customized design of material parameters. In terms of material preparation and industrialization, lab-scale synthesis methods such as *in situ* growth and microwave assembly suffer from low yields and inferior batch uniformity. Costly precursors, specialized facilities, and energy-intensive high-temperature pyrolysis (required for enhanced conductivity) restrict industrial manufacturing. Consequently, Si@MOF composites have a far higher overall cost than conventional silicon-carbon (Si/C) materials and thus show weak market competitiveness. As analyzed above, organic ligands are the primary contributor to high precursor costs and the dominant determinant of MOF toxicity, as validated by toxicological research. *In vitro* tests on human bronchial epithelial cells reveal that ZIF-8 (2-methylimidazole linker) possesses a lower IC_50_ and stronger cytotoxicity than carboxylic acid-based MIL-160. A comprehensive review further establishes a clear toxicity ranking: multi-carboxylate ligands for MIL-100 and MIL-88B are less toxic and readily metabolizable; 2-methylimidazole for ZIF-8 is more hazardous; non-biodegradable phenolic conductive ligands (HHTP and DHBQ) present the greatest safety threats. Consistently, MIL materials sustain higher safe *in vitro* concentrations than ZIF-8. Metal centers and material hydrophobicity synergistically intensify cytotoxicity, with hydrophobic ZIF-8 being prone to cellular damage, while surface coating effectively suppresses toxic ligand leaching.^130–132^

For battery deployment, MIL ligands outperform ZIF linkers, and both surpass phenolic conductive ligands in terms of lower toxicity and recyclability. Industrial-grade 4,5-imidazoledicarboxylic acid, trimesic acid and 2-methylimidazole for MIL/ZIF synthesis are cheap and readily available, yet their battery-grade ultra-high-purity counterparts come at a substantial premium. Ultra-pure HHTP and DHBQ suffer from global supply shortages and are biodegradable; real-time prices can be checked *via* relevant platforms. In comparison, metal salt precursors for MIL and ZIF are widely accessible and cost-effective. (Battery raw material price inquiry website: https://www.sigmaaldrich.cn/CN/zh/campaigns/materials-for-battery-rd-manufacturing, the prices of organic ligands (10 g per batch) are listed as follows: trimesic acid (H_3_BTC, CAS: 554-95-0, purity 99.978%), 99.978; imidazole-4,5-dicarboxylic acid (CAS: 570-22-9), 649.84; 2-methylimidazole (CAS: 693-98-1), 55.40; 2,3,6,7,10,11-hexahydroxytriphenylene (HHTP, CAS: 4877-80-9), 768; 2,5-dihydroxy-1,4-benzoquinone (DHBQ, CAS: 615-94-1), 531.553. All quoted prices correspond to a 10 g reagent package).

To address these bottlenecks, this work proposes a combined framework of machine learning (ML) pre-screening and density functional theory (DFT) verification. DFT enables atomic-scale investigation into MOF conductivity, structural stability and redox activity, evaluates calculation errors caused by material defects, and defines the scope of different theoretical models, laying a solid foundation for mechanistic analysis. ML features are categorized into manually designed descriptors and automatically extracted features *via* deep learning.^133,134^ We suggest prioritizing interpretable hand-crafted descriptors for material property prediction, with the model adopting three core parameters: metal node electronegativity, organic ligand length and pore volume. These descriptors separately reflect electronic properties, geometric framework structures and pore characteristics of MOFs, quantifying their electron transport capacity, reversible coordination bond distortion and lithium ion diffusion performance. Meanwhile, we advocate coupling the Molecular Graph Transformer with the SHAP algorithm for high-throughput screening of large databases to eliminate black-box prediction.^135,136^

Reported state-of-the-art research in this field is summarized as follows: in the field of MOFs, conventional ML paradigms such as Crystal Graph Convolutional Neural Network (CGCNN) and descriptor-based models only capture either local atomic chemical features or global geometric pore descriptors in isolation, which restricts their cross-task predictive capacity and requires rebuilding models from scratch for every property prediction task. To address this limitation, a multi-modal pre-training transformer framework named MOFTransformer is proposed, which implements universal Transfer Learning (TL) *via* a two-stage training pipeline: pre-training on one million hypothetical MOFs (hMOFs) through three self-supervised auxiliary tasks (MOF topology prediction (MTP), void fraction prediction (VFP), and metal/organic linker classification (MOC)), followed by lightweight fine-tuning on small datasets of merely 5000–20 000 MOF structures.^137^ Mechanistically, the model fuses two complementary embedding branches simultaneously: atom-based graph embedding extracts local chemical information from metal nodes and organic linkers, while energy-grid patch embedding encodes global topological and pore energy landscape features; the bidirectional transformer encoder equipped with multi-head self-attention layers integrates multi-scale structural signals and quantifies feature weights *via* attention scores to interpret the underlying structure–property relationships (SPR). The integrated multi-modal feature fusion mechanism resolves the intrinsic defect of single-feature representation, where diffusion behaviors dominated by global void geometry gain high attention weights on energy-grid embeddings, whereas electronic band gaps governed by orbital coupling of atomic building blocks rely heavily on local graph embeddings. Experimental results demonstrate that MOFTransformer achieves State-of-the-Art (SOTA) prediction accuracy across diverse downstream tasks including molecular diffusivity, electronic band structure, and text-mined material stability data, delivering higher *R*^2^ and lower mean absolute error (MAE) than the CGCNN, energy histogram baselines and other traditional ML models even when fine-tuned on limited training samples. This interpretable universal pre-training architecture drastically reduces data and computational costs for SPR exploration and provides quantitative chemical guidance for rational MOF design. However, few studies have leveraged machine learning for Si@MOF screening, which underscores the cutting-edge characteristic of this field.^137–139^

This integrated framework resolves three key bottlenecks. ML coupled with a molecular graph transformer quantifies Si-MOF pore-matching thresholds, predicts HOMO/LUMO levels to correlate metal coordination with SEI interfacial stability, and constructs a precursor-pyrolysis-conductivity quantitative model for accurate experimental regulation. DFT-ML collaboration drastically cuts screening duration and costs. This strategy supports the design and mechanistic study of breathing MOF/Si composite anodes, promotes quantitative electrode development, and provides solutions for Si@MOF preparation and industrialization.^135,140^

**Fig. 16:**
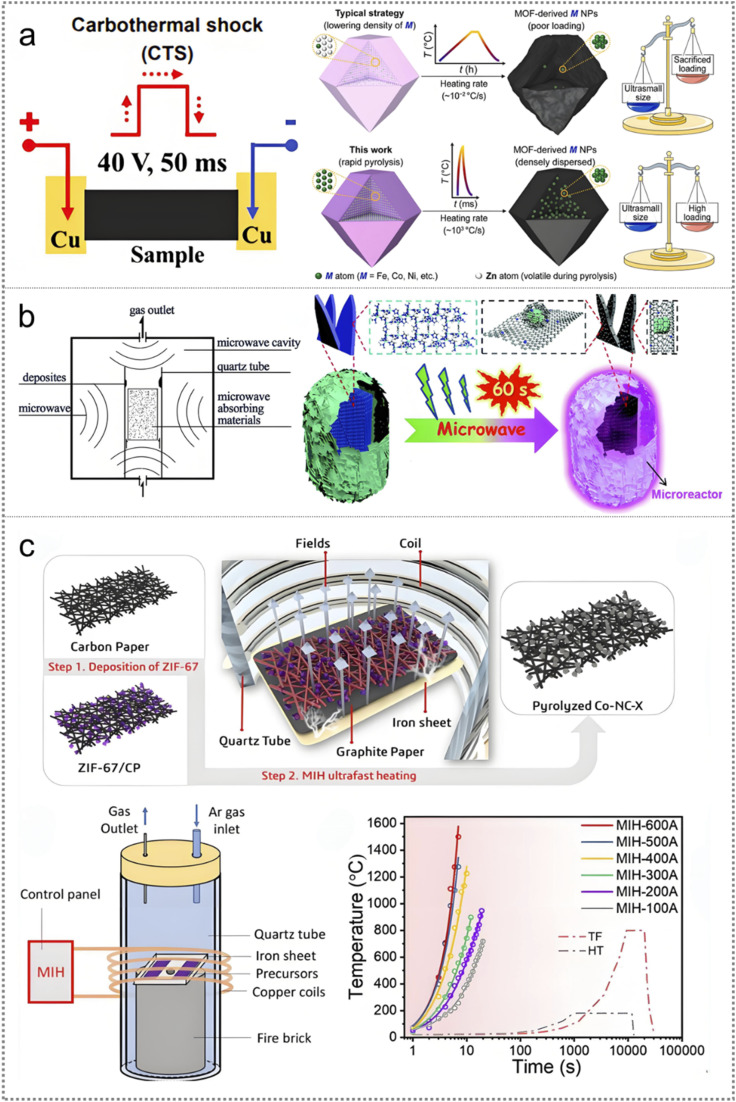
Three ultrafast pyrolysis techniques for MOF-derived ultrasmall M nanoparticles.^141^ Adapted/reproduced from ref. 141 with permission from Elsevier, copyright 2024. (a) CTS: 40 V per 50 ms electroheating forms high-loading, highly dispersed M nanoparticles. (b) Microwave pyrolysis: 60 s synthesis of microreactor-structured M nanoparticles. (c) MIH: Ultrafast heating of ZIF-67 on carbon paper to produce Co-NC-X, with a verified superior heating rate.

Given the inherent limitations of Si@MOF-derived composites in conventional thermal treatment processes, emerging technologies like rapid Joule heating (RJH), laser-induced heating (LIH), and microwave heating (MWH) are viable to address high energy consumption and long cycles in traditional preparation. Boasting high energy efficiency and ultra-fast reaction kinetics, they enable greener and more efficient preparation: RJH achieves second-scale reactions with energy consumption as low as kJ g; LIH uses nanosecond heating/cooling and up to 4000 K temperature control to ensure product uniformity and shorten cycles; MWH leverages selective heating to cut energy consumption by 99.6% *vs.* traditional pyrolysis and boost yield to 48.7%. Fig. 16 illustrates the ultrafast pyrolysis synthesis of highly dispersed ultrasmall MOF-derived metal NPs; (1) carbothermal shock (CTS): 40 V per 50 ms electroheating (∼10^4^ °C s^−1^, *vs.* conventional ∼10^−2^ °C s^−1^) rapidly pyrolyzes MOFs, volatilizes Zn, and *in situ* forms ultrasmall, high-loading, highly dispersed M NPs (Fig. 16a); (2) microwave-assisted pyrolysis: 60 s microwave heating converts MOF precursors into microreactor-structured M NPs (Fig. 16b); (3) microwave-induced heating (MIH): ultrafast heating of ZIF-67 on carbon paper yields Co-NC-X, with superior heating rates (*vs.* traditional tube furnace/hydrothermal methods) verified by device and temperature curves (Fig. 16c). These technologies overcome traditional energy efficiency and cycle bottlenecks, supporting large-scale green production of Si@MOF derived composites.^141^

Beyond the aforementioned structural limitations, insufficient quantitative exploration and mechanistic cognition of MOF dynamic breathing behavior remains a crucial bottleneck restricting the practical application of Si@MOF composites. Various spectroscopic and microscopic tools can be adopted to characterize the breathing behavior of flexible MOF shells in Si@MOF anodes for lithium-ion batteries. *Operando* PXRD quantifies reversible lattice expansion/contraction of MOFs to reveal how their elastic breathing buffers huge silicon volume fluctuation during lithiation and delithiation. *Operando* XAS (EXAFS and XANES) tracks atomic-scale reversible distortion of metal–ligand coordination bonds under cyclic electrochemical stress. *In situ* Raman and FTIR capture ligand torsional vibrations induced by framework breathing, while MAS solid-state NMR evaluates the structural recoverability and fatigue resistance of flexible MOF scaffolds after long cycling. DMA further measures the framework elasticity and cyclic deformation tolerance to explain the long-cycle stability of Si-based electrodes, and environmental TEM directly visualizes dynamic pore shrinkage/expansion of MOF coatings on silicon particles.^142^

Finally, in terms of commercial applications. A critical practical drawback of MOF-based silicon anodes lies in their abundant intrinsic pores: while such voids effectively buffer silicon's lithiation-induced volume expansion, excessive porosity drastically reduces electrode tap density and volumetric energy density. Breathable MOFs achieve improved gravimetric capacity at the cost of sharply reduced volumetric performance relative to dense commercial Si/C composites. This trade-off fails to meet the core demands of electric vehicles, which face strict spatial limits and rely on high volumetric energy density to extend the driving range. To balance strain-buffering voids and high electrode tap density, three typical optimization strategies are summarized as follows: (1) bimodal particle packing: mono-sized MOF powders form massive interparticle voids and exhibit low volumetric capacity. Matching micro- and nanosized identical MOF particles allows fine nanoparticles to fill gaps between large particle skeletons, increasing packing density without destroying intrinsic pores. For V3(PET, H6PET = 4,4′,4″,4‴,4‴′,4‴″-(9,10-dihydro-9,10-[1,2]benzenoanthracene-2,3,6,7,14,15-hexayl)hexabenzoic acid) MOFs, blending 9 µm and 300 nm crystals raises the packing fraction from 0.42 to 0.56 and boosts volumetric capacity by 33–38%. MOF-5 tests further prove that bimodal cubic crystals achieve high packing density while preventing framework collapse under compression.^143^ (2) Dense conjugated hierarchical porous frameworks: conventional MOFs rely on excessive loose pores for Li storage and thus form fluffy powders. This interpenetrated rigid 3D skeleton retains only limited ultramicropores for Li^+^ transport and minor mesopores for strain relief, while dense lithiophilic sites (Fe centers, O groups, and benzene rings) guarantee high capacity without extra porosity. The Fe-Tp anode reaches 1447 mAh g^−1^; doping 10 wt% Fe-Tp into graphite doubles cycle capacity and offsets tap density loss by filling graphite interstices. The design strategy of dense conjugated frameworks can be adopted for silicon Si SAs or silicon crystallites covered in this review.^144^ (3) Ultrathin compact MOF interfacial coating: sub-40 nm MOF films grown on substrates occupy little volume. The dense shell guides uniform Li deposition, suppresses dendrite-induced fluffy Li layers, and reduces internal electrode voids, while reserved micropores sustain ion conduction and strain accommodation. Layer-by-layer Ag-TCA ultrathin MOF coating on textile Li anodes generates *in situ* Ag lithiophilic sites to homogenize Li plating. The LiFePO_4_ full cell retains ∼96.5% capacity over 1300 cycles at 1C, validating balanced interfacial stability and volumetric utilization.^145^ In brief, bimodal packing optimizes powder stacking at the macroscopic scale, dense hierarchical pore skeletons adjust crystal pore structures at the microscopic scale, and ultrathin MOF coating regulates particle interfaces. All three strategies verify that gradient pore design and densified MOF modification effectively resolve the trade-off between strain-buffering porosity and high volumetric energy density.^146^

**Fig. 17:**
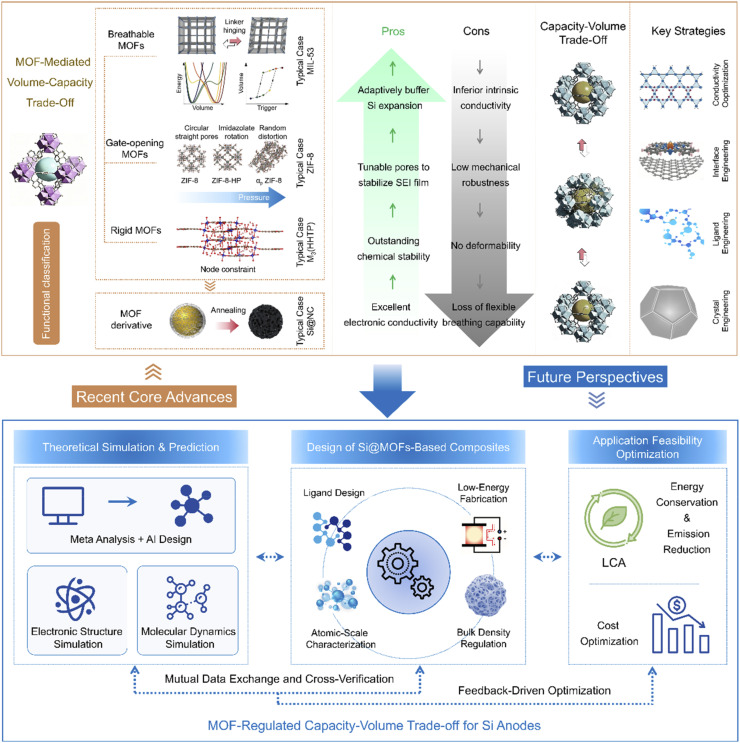
Current progress and future perspectives.

Notably, most existing electrochemical evaluations of Si@MOF anodes are limited to low-mass-loading half-cell configurations,^67,100^ while systematic applicability assessments under high-loading electrode conditions and rigorous full-cell/flexible pouch-cell validations remain scarce, which restrict objective assessment of their practical industrialization potential. Accordingly, we highlight the urgent need for more full-cell and pouch-battery validation experiments to reliably evaluate real-world application prospects. In particular, the three optimized strategies summarized in this work—bimodal particle packing, dense conjugated hierarchical pore framework design, and ultrathin compact MOF interfacial coating—can be further verified under full-cell/pouch-cell working conditions to advance the scalable deployment of MOF-modified silicon anodes (Fig. 17).

## 6. Conclusions and prospects

Silicon anodes have excellent capacity but suffer from significant volume expansion, limiting their practical application. Compared with traditional modification methods, MOFs enable the synergistic balance between silicon's high capacity and structural stability *via* multiple effects (stress absorption, SEI regulation, electron transmission promotion, *etc*). Existing Si@MOF composite strategies lay a foundation for performance optimization.

Several key issues remain unsolvable systematically, mainly in the following three aspects: (1) systematic regulation of MOF metal nodes, ligands, and coordination bond lengths is challenging, without a quantitative correlation with silicon anode characteristics; (2) the matching mechanism of MOF size, silicon particle size and electrode pore structure is unestablished, with inappropriate pore sizes affecting silicon loading, agglomeration and lithium-ion intercalation; (3) the stress between MOFs and silicon anodes is hard to precisely match, where insufficient stress fails to buffer volume expansion, while excessive stress suppresses their “breathing” properties.

To address the critical issue of capacity–expansion trade-off, future research should focus on the above key issues and directions to advance the practical application of Si@MOF composites and provide new paths for next-generation high-performance lithium-ion batteries:

(i) establishing node–ligand compatibility regulation (*i.e.*, correlation between stress trigger and MOF contraction threshold) *via* machine learning and theoretical calculations.

(ii) Developing a low-cost, high-yield, non-destructive encapsulation strategy to narrow the laboratory–industrial gap.

(iii) By integrating *in situ* structural characterization, mechanical-electrochemical coupling tests, and multi-scale theoretical simulations in a multi-dimensional research framework, we aim to deeply explore the mechanism of the dynamic breathing effect of MOFs and its correlation with electrochemical performance, in order to promote their application in high-energy-density lithium-ion batteries.

## Author contributions

Wenhao Zhou: conducted research and verification, completed manuscript drafting and figure plotting, and performed subsequent article revisions. Fan Mo: conducted research and validation, revised the manuscript, prepared figures, and oversaw overall supervision of the study. Yitong Wang: conducted systematic research. Xinrui Mu: conducted systematic research. Haibo Li: provided conceptual suggestions, oversaw the entire project and offered research-related guidance.

## Conflicts of interest

The authors declare that they have no competing financial interests or personal relationships that could have appeared to influence the work reported in this paper.

## Data Availability

No primary research results, software or code have been included and no new data were generated or analysed as part of this review.
